# Energy‐Efficient, Sustainable Cascade Glucose Electrooxidation into Glucaric Acid

**DOI:** 10.1002/adma.202519531

**Published:** 2025-12-24

**Authors:** Mingming He, Chao Huang, Mingzi Sun, Ruixuan Wang, Yun Song, Jianjun Su, Weihua Guo, Yinger Xin, Qiang Zhang, Yong Liu, Geng Li, Zihao Li, Rui Xue, Bolong Huang, Ben Zhong Tang, Ruquan Ye

**Affiliations:** ^1^ Department of Chemistry and State Key Laboratory of Marine Environmental Health City University of Hong Kong Hong Kong P. R. China; ^2^ State Key Laboratory of Pulp and Paper Engineering South China University of Technology Guangzhou P. R. China; ^3^ Guangdong Basic Research Center of Excellence for Aggregate Science School of Science and Engineering The Chinese University of Hong Kong (Shenzhen) Shenzhen P. R. China; ^4^ Department of Chemistry and the Hong Kong Branch of Chinese National Engineering Research Center for Tissue Restoration and Reconstruction The Hong Kong University of Science and Technology Hong Kong P. R. China; ^5^ City University of Hong Kong Shenzhen Research Institute Shenzhen China

**Keywords:** biomass valorization, energy saving, glucaric acid, glucose electrooxidation, tandem electrolysis

## Abstract

Glucaric acid (GRA) is a critical platform chemical for manufacturing biodegradable materials. Selective glucose (GLU) electrooxidation into GRA provides a sustainable route for biomass valorization. However, conventional methods suffer from energy‐intensive processes due to excessive operational potential exceeding 1.2 V. Here we demonstrate an energy‐efficient tandem system that decouples GRA electrosynthesis into cascade GLU‐to‐gluconic acid (GNA) and GNA‐to‐GRA oxidation. When pairing an Au/C catalyst for selective aldehyde oxidation and an AuPt/C catalyst for hydroxyl oxidation, we achieve 91.8% Faradaic efficiency and nearly 100% conversion efficiency at 0.6 V_RHE_ for GLU‐to‐GNA oxidation, and 81% Faradaic efficiency and 90% conversion efficiency at 0.55 V_RHE_ for GNA‐to‐GRA oxidation. Chronoamperometry demonstrates ∼100% substrate conversion with a minor decrease in product selectivity, confirming the catalyst's excellent stability. Our tandem system improves the overall GLU‐to‐GRA energy efficiency from 13.8% for conventional one‐step route to 31.8%. When oxygen reduction is selected as paired reaction, our system not only enables efficient chemical electrosynthesis, but is also estimated to generate electricity of 1.24 × 10^5^ kWh per kiloton GRA, outperforming traditional method with energy consumption of 4.31 × 10^5 ^kWh. Our work establishes a sustainable and economically viable pathway for biomass valorization, offering a blueprint for circular, carbon‐neutral chemical production.

## Graphical abstract

This study presents a tandem system that efficiently converts glucose into glucaric acid while generating renewable electricity. By decoupling the oxidation process into two cascaded steps, the system achieves an overall 90% Faradaic efficiency and 80% conversion efficiency at ∼0.6 V_RHE_, significantly reducing energy consumption compared to traditional one‐step routes at >1.2 V_RHE_.

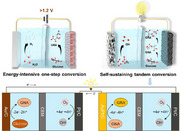

## 1 Introduction

Biomass represents a renewable and environmentally benign alternative to fossil‐fuel‐derived feedstocks for chemical production, offering a sustainable pathway to mitigate pressing environmental challenges [1, 2, 3, 4, 5]. Among biomass‐derived platform molecules, GLU stands out due to its well‐defined oxidation chemistry, which can be tailored to produce high‐value products, including 5‐hydroxymethylfurfural, sorbitol, gluconic acid (GNA), and glucaric acid (GRA) [6, 7, 8]. Notably, GRA has been designated by the U.S. Department of Energy as a “top‐value‐added chemical” owing to its versatile applications, ranging from biodegradable polymers and detergents to food additives and pharmaceuticals [9, 10, 11]. With its expanding applications, the global GRA market is poised for substantial growth in the coming decades.

**FIGURE 1:**
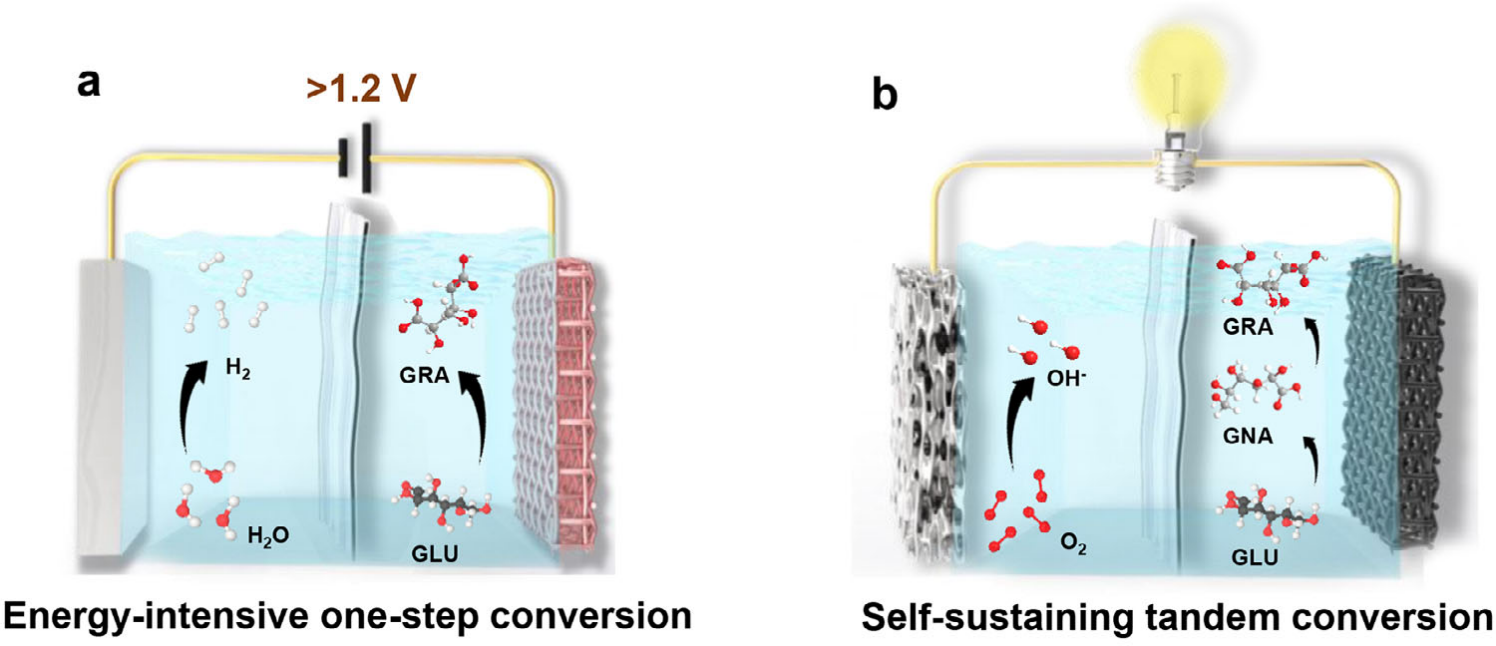
Schematic diagram of (a) one step and (b) two‐step tandem process for the electrocatalytic production of GRA from GLU.

Current methods for GLU oxidation to GNA and GRA predominantly employ chemical catalysis, microbial fermentation, or single‐step electrochemical approaches [12, 13]. However, chemical and biological methods face significant limitations, including the use of toxic oxidants, prolonged fermentation times (>48 h), and poor selectivity (<20% GRA yield) [14, 15, 16]. In contrast, electrochemical oxidation has emerged as a promising alternative that operates without hazardous reagents or high oxygen pressures [17, 18, 19, 20, 21]. This method enables precise selectivity control through potential modulation, effectively minimizing byproduct formation [22, 23, 24, 25]. Notably, Qiao et al. [26]. demonstrated a defect‐rich FeCoNiCu catalyst that achieved 100 mA cm^−2^ at 1.22 V vs. reversible hydrogen electrode (RHE; all potentials are referenced to RHE unless specified) with >90% GRA yield. However, such transition metal systems typically require potential of >1 V, resulting in considerable energy demands (Figure 1a). The fundamental challenge in GLU‐to‐GRA conversion lies in the distinct oxidation potentials of aldehyde (C_1_ position) and hydroxyl (C_6_ position) groups [27, 28]. For example, while gold (Au) catalysts efficiently oxidize the aldehyde group below 1 V, the C_6_ hydroxyl oxidation requires significantly high potentials (∼1.3 V) [29, 30]. This energetic mismatch leads to substantial efficiency losses during direct one‐pot conversion.

In stark contrast to the conventional one‐step GLU oxidation, here we present a paradigm‐shifting tandem electrochemical system that decouples aldehyde and hydroxyl oxidation steps to simultaneously achieve product upgrading and energy recovery (Figure 1b). Our approach leverages: (1) initial GLU‐to‐GNA conversion using an optimized Au/C catalyst (initial oxidation potential: 0.3 V), and (2) subsequent GNA‐to‐GRA oxidation with a AuPt/C catalyst initiating at 0.55 V. Since the thermodynamic redox potential of O_2_/OH^−^ is 1.23 V and some catalysts have achieved a half‐wave potential of 0.95 V, by strategically pairing these reactions with oxygen reduction, the GLU‐to‐GRA conversion will become energetically self‐sustaining. Experimentally, we demonstrate that in the first step, nearly 100% GLU can be converted with a Faraday efficiency (FE) of 91.8% for GNA. In the second step, we achieved the further oxidation of GNA to GRA with an 81% FE and 80% conversion efficiency. Thus, by utilizing the electron from GLU‐to‐GNA oxidation to drive the GNA‐to‐GRA conversion, this tandem configuration transforms the conventional energy‐intensive process into an electricity‐generating system while maintaining high product selectivity, establishing a new benchmark for sustainable biomass valorization.

## 2 Results and Discussion

### 2.1 The Conversion of GLU to GNA

In the proposed unassisted GRA synthesis reaction pathway, two steps are involved: (1) oxidation of GLU to GNA, which is a two‐electron‐transfer process, and (2) oxidation of GNA to GRA, which involves four electrons transfer (Figure S1). Hence, GNA is the key bridging intermediate that facilitates the oxidation of GLU to GRA. However, some side reactions may occur in this process, such as the isomerization of GLU, C─C bond cleavage, and overoxidation, which reduce the selectivity of GNA and produce undesired by‐products such as formic acid (HCOOH), glucuronic acid (GLA), glycolic acid (GA), and tartaric acid (TA). Therefore, identifying an optimal catalyst with low overpotential and high selectivity is crucial to improve the selectivity and energy efficiency of GLU‐to‐GNA conversion.

The anodization of GLU can be catalyzed by Au, palladium (Pd), platinum (Pt), copper (Cu), nickel (Ni), and iron (Fe) based catalysts [31, 32, 33]. Thus, we started with the screening of catalyst candidates in achieving selective GLU‐to‐GNA conversion at low overpotentials. We prepared Au/C, Pt/C, Pd/C, Cu foam (abbreviated as Cu), and nickel iron hydroxide (abbreviated as NiFe) as the catalysts. The transmission electron microscope (TEM), energy‐dispersive X‐ray spectroscopy (EDS) image (Figures S2–S7), and X‐ray diffraction (XRD, Figures S8–S13) confirmed the successful synthesis of these catalysts. For instance, the Au/C sample shows the presence of 38.18°, 44.39°, 64.57°, and 77.54° peaks in XRD, which correspond to the (111), (200), (220), and (311) crystal facets of Au (Figure S8). TEM images show the uniform size distribution of ∼30 nm and the characteristic Au (200) and (111) facets (Figure S2b,c). X‐ray photoelectron spectroscopy (XPS) analysis indicates that Au in the sample is in the zero‐valence state (Figure S13).

**FIGURE 2:**
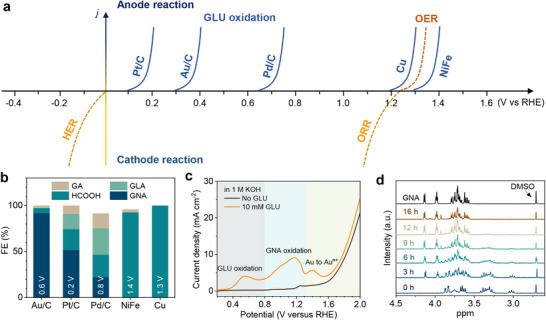
The conversion of GLU to GNA. (a) Comparison of GLU oxidation potential on different catalysts. (b) Selectivity of GLU oxidation on various catalysts. The applied potential is indicated in each column. The experiment was conducted in 0.01 M GLU + 1 M KOH. (c) LSV curves of Au/C electrode in 1 M KOH, with (red curve) and without (black curve) 10 mM GLU. (d) ^1^H spectra of GLU electrooxidation products versus time on Au/C catalyst.

We first compare the electrochemical activities of GLU oxidation by testing the linear scanning voltammetry (LSV) curves (Figure S14), and Figure 2a summarizes the reaction onset potentials of all catalysts in 0.01 M GLU solution. The oxidation potential of GLU oxidation is in the order of NiFe > Cu > Pd/C > Au/C > Pt/C. The oxidation potential for noble metal‐based catalysts such as Pt/C, Au/C, and Pd/C are all less than 1 V, whereas the non‐noble metal‐based catalysts Cu and NiFe start at potentials greater than 1.2 V. Considering the four‐electron oxygen reduction reaction (ORR) has a thermodynamic potential of 1.23 V, pairing noble metal‐based catalysts with ORR will be an energy‐releasing process. We then evaluate the product selectivity of different catalysts near the oxidation potential (Figure 2b). Au/C is the most favorable catalyst to oxidize the aldehyde group of GLU to carboxylic group with an FE_GNA_ as high as 91.8%. Although the oxidation potential of GLU oxidation of Pt/C is lower than that of Au/C, the selectivity of GNA is only 51.7%, much less than that of Au/C. In stark contrast, the non‐noble metal‐based catalysts mainly yield HCOOH due to the favorable C─C bond cleavage at high overpotentials. Therefore, we further study the Au/C catalyst to understand the unique performance of Au/C catalyst in oxidizing GLU into GNA.

To investigate the catalytic activity of Au/C for GLU oxidation, the LSV curves (Figure 2c) of the Au/C catalyst in 1 m KOH solution with and without GLU were tested. The addition of 10 mM of GLU results in apparent anodic peaks at 0.53, 1.15, and 1.4 V, respectively. Since the peak at 0.53 V disappears in 10 mm GNA solution (Figure S15), it can be inferred that the 0.53 V peak belongs to the oxidation of the aldehyde group of GLU, and the 1.15 V peak is related to the oxidation of GNA. In 1 m KOH solution, the peak at 1.25 V corresponds to the electrooxidation of Au [30]. When 10 mm GLU solution is added, the oxidation peak of Au shifts to a higher potential of 1.4 V, which can be attributed to the adsorption of organic molecules on the catalyst surface. ^1^H nuclear magnetic resonance (NMR, Figure 2d) was employed to comprehensively monitor the conversion of GLU, and chronoamperometry measurements were conducted at a constant potential of 0.6 V. Figure 2d reveals a gradual decreasing of GLU's characteristic peaks at 2.9–3.4 ppm, alongside a corresponding strengthening of the GNA peaks at 3.9–4.3 ppm, confirming the successful oxidation of GLU to GNA. After 12 h, the conversion of GLU is almost 100%, and the FE of GNA reaches 91.8%. The influence of GLU concentrations on GLU oxidation was investigated. Figure S16 demonstrates that the current density increases with increasing GLU concentrations. When the GLU concentration is in the range of 30–180 mm, the current density at E = 0.6 V is in linear relationship to the GLU concentrations (Figure S16b), whereas the current density remains unchanged when the GLU concentration further increases up to 470 mm. This implies that electrochemical GLU oxidation matches the first‐order reaction kinetics at low GLU concentrations [31].

In addition to activity, we further evaluated the stability and durability of Au/C for the GLU electrooxidation. We first performed five chronoamperometry cycles consecutively (Figure S17a). The results reveal that the conversion of GLU remains nearly 100% and the FE of GNA ranges from 91.8% to 86.78% (Figure S17b). Moreover, the long‐term stability of the GLU electrooxidation was evaluated. As shown in Figure S17c,d, the electrooxidation process can operate continuously for 15 h at 0.6 V, maintaining 84% of its original current density and FE_GNA_. To further evaluate the structural stability of the Au/C electrocatalyst, scanning electron microscope (SEM), TEM, XRD, XPS, and inductively coupled plasma (ICP) were used to fully characterize the electrocatalyst. SEM and TEM patterns show that the spherical particle structure of the reused Au/C remains unchanged (Figure S18a,b). The XRD pattern of the post‐reaction Au/C (Figure S19) shows the major peaks of Au. The XPS spectra for Au/C show negligible change in peak position of Au, indicating that valence states remain unchanged after the reaction (Figure S13b,c). The ICP analysis of the electrolyte reveals negligible metal dissolution during the oxidation process. These findings demonstrate the excellent stability of the Au/C catalyst, suggesting good durability for long‐term use.

### 2.2 The Conversion of GNA to GRA

The direct oxidation of GLU to GRA typically requires high potential and the GRA yield is low due to the excessive oxidation [31]. In our concept, oxidizing from GNA to GRA presents several advantages. Specifically, the decoupled oxidations allow for optimizing catalysts that selectively oxidize aldehyde and hydroxyl groups. Since the aldehyde and hydroxyl groups proceed at different oxidation potentials, the cascade process can reduce the overall applied potentials and minimize the energy costs. The reduced applied potential also alleviates the excessive GLU oxidation issues, which helps improve the GRA yield.

**FIGURE 3:**
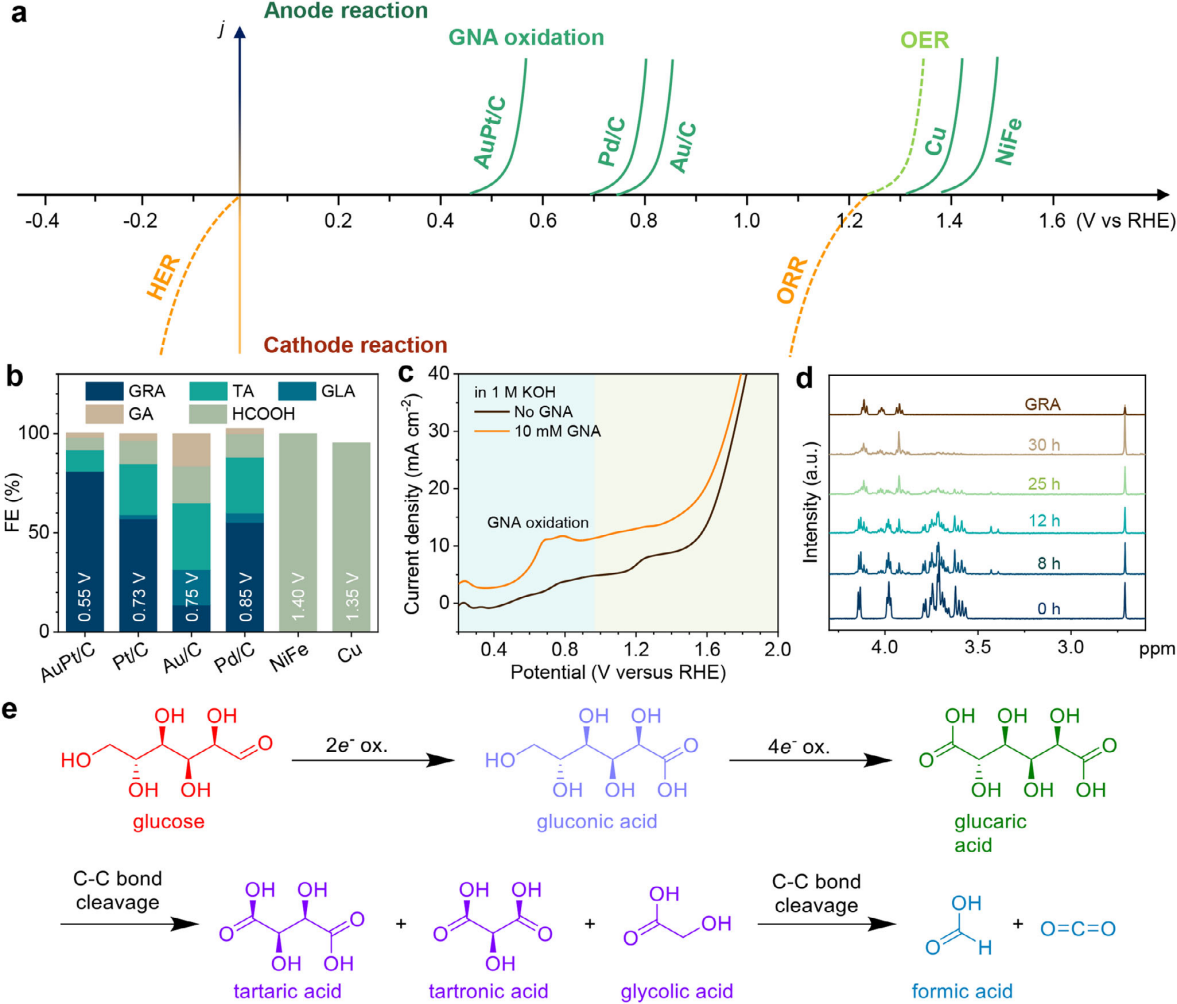
The conversion of GNA to GRA. (a) Comparison of GNA oxidation potentials on different catalysts. (b) Selectivity of GNA oxidation on various catalysts. (c) LSV curves of AuPt/C electrode in 1 M KOH, with (green) and without (yellow) 10 mM GLU. (d) ^1^H spectra of GNA electrooxidation products versus time on AuPt/C catalyst. (e) Schematic of different GLU oxidation pathways.

To identify an optimal catalyst for the conversion of GNA to GRA, we conducted a comprehensive screening of a variety of catalysts, including Pt/C, Pd/C, Au/C, Cu_2_O, and NiFe. Likewise, we first compare the GNA oxidation potential (Figure S23), and the results are summarized in Figure 3a. Similar to the GLU oxidation, the GNA oxidation potential is less than 1 V for noble metal‐based catalysts such as Pt/C, Au/C, and Pd/C, while the non‐precious metal‐based catalysts Cu_2_O and NiFe exhibit high oxidation potential. The superior performance of noble metals in catalyzing GNA oxidation can be attributed to their unique electronic structures, which confer excellent adsorption properties for organic molecules. For instance, Pt has a *d*‐electron configuration that allows for efficient absorption and activation of reactant molecules. The interaction between Pt's d‐orbitals and the molecular orbitals of GNA weakens the key bonds such as the C─H and C─O bonds, thereby lowering the energy barrier for the oxidation reaction and leading to a lower oxidation potential [34].

Although Au/C exhibits excellent performance in the GLU‐to‐GNA conversion, its activity for GNA oxidation remains suboptimal. The oxidation potential for GNA‐to‐GRA is nearly 0.8 V for Au/C (Figure S15), which indicates that high energy is required to initiate the reaction. In contrast, Pt/C catalyst exhibits a lower oxidation potential for this transformation but is susceptible to poisoning, limiting its practical application [35, 36]. Literature studies suggest that alloying Pt with a second metal can enhance its catalytic activity, resulting in higher stability and activity than pure Pt [37, 38]. Therefore, we synthesized the AuPt/C alloy catalyst, aiming to reduce the oxidation potential, enhance the selectivity of GRA, and mitigate the poisoning issues of Pt.

Figure 3b compares the GNA oxidation performance of different catalysts at their anodic peaks determined from CV curves. Among different catalysts, precious metals outperform non‐precious ones. Specifically, AuPt/C catalyst has the highest selectivity for GRA with a FE of 81%, which is better than Pt/C (57%) and Au/C (14%). Notably, NiFe and Cu_2_O catalysts oxidize GNA at higher potentials and they predominantly generate HCOOH with >90% FE due to excess oxidation. Considering both applied potential and product selectivity, we select AuPt/C as the catalyst for the second‐step GNA‐to‐GRA conversion.

The LSV curves of AuPt/C catalysts were conducted with and without the addition of GNA in 1 m KOH solution to further investigate the catalytic activity of AuPt/C for GNA oxidation. As shown in Figure 3c, in the presence of GNA, a distinct anodic peak started at 0.5 V RHE, which can be attributed to the oxidation peak of GNA. The oxidation potential of AuPt/C is the lowest for GRA production among the reported data (Table S1). The AuPt/C catalyst attains a current density of 10 mA cm^−2^ at 0.66 V. Similar to the first step, the effect of initial GNA concentration was studied. Figure S24 illustrates the current density response to varying GNA concentrations from 0.01 to 0.34 m. The current density increases significantly as the GNA concentration rises to 0.13 m, after which the increments diminish. This initial enhancement is primarily due to the increased availability of reactants on the active surface. At higher concentrations, the current density is limited due to the saturation of reaction sites.

NMR (Figure 3d) and high‐performance liquid chromatography (HPLC, Figure S25) were employed to comprehensively monitor the conversion of GNA, and chronoamperometry measurements were conducted at a constant potential of 0.55 V. Figure 3d illustrates that as the reaction progresses, the characteristic peaks of GNA at 3.5–3.8 ppm diminishes, while those of GRA at 3.8–4.2 ppm become intensified, confirming the successful oxidation of GNA to GRA. After 36 h, the conversion of GNA approaches 90%, and the FE of GRA reaches 81%. Compared to GLU oxidation, the lower oxidation rate of GNA is due to the sluggish kinetics of the dehydrogenation process from ‐CH_2_OH to ‐CHO, a critical step in GNA activation that is not required for the oxidation of GLU to GNA [28]. We also notice that the selectivity of GNA‐to‐GRA conversion is inferior to that of GLU‐to‐GNA. This is probably because of the higher applied potential that initiates some side reactions, as shown in Figure 3e. The higher applied potential can lead to C─C bond cleavage, eventually forming C_1_, C_2,_ and C_4_ side products.

XPS and XRD were utilized to characterize the structure of AuPt/C. As shown in Figure S26a, the 4f binding energies of Au in AuPt/C are 84.48 and 88.14 eV, indicating the presence of both metallic Au (Au (0)) and oxidized Au (Au (III)) [39]. The Pt 4f binding energies are 70.88, 74.16, 71.71, and 74.93 eV, respectively, indicating that the Pt presence is dominated by the Pt (0) and Pt (II) valence states. Compared to the pure samples shown in Figure S26c,d, the binding energies of Au 4f in AuPt/C shift slightly higher by approximately 0.1–0.2 eV, while those of Pt 4f become smaller by a similar margin. These shifts in XPS peaks may indicate a slight electron transfer from Au to Pt [40]. XRD analysis reveals that the peaks of AuPt/C appeared at 38.7° (111), 44.9° (200), positioned between the peaks of Pt/C and Au/C catalysts, implying the formation of alloy. The high‐resolution TEM (HRTEM) image (Figure S28c) of AuPt/C indicates that the d spacing between neighboring lattice planes was determined to be 0.231 nm, which is between the (111) planes of fcc Au (0.2355 nm) and Pt (0.2265 nm), in agreement with the XRD results. These observations suggested the formation of single‐phase bimetallic catalysts on the carbon supports [41]. Therefore, the enhanced activity of AuPt/C in the catalytic oxidation of GNA can be attributed to the modulated electronic structure due to alloy formation, which changes reaction energies and enhances the catalytic activity [42].

We also examined the stability and durability of AuPt/C for GNA oxidation by running five chronoamperometry cycles (Figure S30a). AuPt/C shows a slight decrease in the conversion of GNA from 90% to 86%, and a slight decrease in the FE of GRA from 81% to 78% (Figure S30b). The long‐term stability of the GNA electrooxidation was evaluated via chronoamperometry (Figure S30c), which shows that the electrooxidation can operate continuously for 15 h at a voltage of 0.55 V while maintaining more than 80% of the initial current density and FE_GRA_ (Figure S30d). The post‐reaction SEM (Figure S31a) and TEM image (Figure S31b) indicate that the spherical nanoparticles remain well‐dispersed with almost no change compared to the fresh catalyst. The XRD (Figure S27b) indicates that the preservation of characteristic (111) and (200) peaks of the AuPt/C catalyst. As illustrated in Figure S26a–d, the Au 4f and Pt 4f XPS spectra of AuPt/C exhibit no shift after the GNA electrooxidation. Furthermore, ICP tests display negligible metal ion concentrations in the post‐reaction solution (<0.5 ppm). These results suggest the excellent structural stability of AuPt/C catalyst.

### 2.3 Cascade GLU‐to‐GNA and GNA‐to‐GRA conversion

**FIGURE 4:**
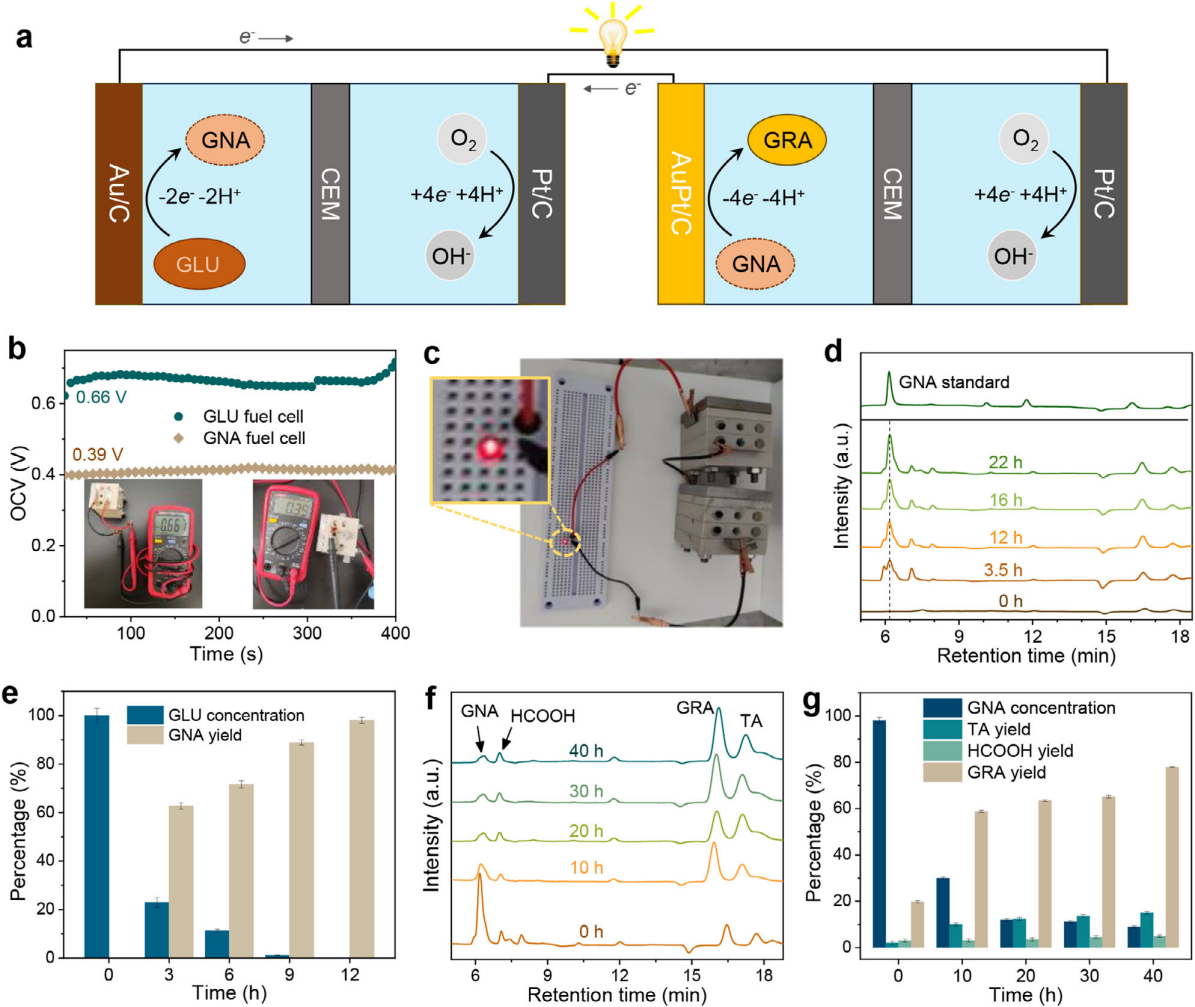
Performance of cascade oxidation of GLU to GNA and GNA to GRA. (a) Schematic of the flow of electrons and ionic charges in a GLU/GNA fuel cell. (b) Open‐circuit voltage of a GLU and GNA fuel cell and (c) Photograph of the GLU fuel cell and GNA fuel cell in series to light a small light bulb. (d) HPLC chromatograms for the GLU oxidation at different reaction times on Au/C catalyst. (e) Concentration of GLU and GNA as a function of time for chronoamperometric tests at 0.6 V vs. RHE. (f) HPLC chromatograms for the GNA to GRA at different reaction times on AuPt/C catalyst and (g) concentration of products as a function of time for chronoamperometric tests at 0.55 V vs. RHE.

To validate the practical application potential of our self‐sustaining biomass upgrading concept, we constructed two biofuel cells (Figure 4a). The first is a GLU fuel cell with Au/C as the anode and Pt/C as the cathode, where GLU is oxidized at the anode and oxygen is reduced at the cathode. The anodic and cathodic electrolytes are 0.3 M GLU + 1 M KOH and 1 M O_2_‐saturated KOH, respectively. The anodic and cathodic reactions are as follows:

(1) Anode:C6H12O6+2OH−→C6H12O7+H2O+2e−

(2) Cathode:1/2O2+H2O+2e−→2OH−

(3) Overall:C6H12O6+1/2O2→C6H12O7

The polarization behavior and power density curves of the cell are presented in Figure S32, showing a maximum power density of 0.89 mW cm^−^^2^ at 0.37 V, with an open‐circuit voltage (OCV) of 0.66 V (Figure 4b). The discharge performance of the cell was investigated (Figure S33). The gradual decay of the current after 24 h of discharge at a constant voltage of 0.37 V leads to a final GNA yield of 50.49%. These experiments demonstrated the feasibility of generating GNA without the need of external electrical energy.

In a similar approach, a GNA fuel cell was constructed with AuPt/C as the anode to facilitate the oxidation of GNA to GRA. The reaction equations are shown below:

(4) Anode:C6H12O7+4OH−→C6H10O8+3H2O+4e−

(5) Cathode:O2+2H2O+4e−→4OH−

(6) Overall:C6H12O7+O2→C6H10O8+H2O

As illustrated in Figure 4b, the GNA fuel cell exhibits an open‐circuit voltage of 0.39 V, indicating that GNA can theoretically be oxidized spontaneously. However, to drive the oxidation of GNA at higher current densities, additional energy input is demanded due to the voltage drop. A viable approach is to connect the two biofuel cells in series. Another possible solution is to temporarily store the biofuel cell energy as electromagnetic energy, as reported by Alex et al. [43]. A boost converter will then amplify the output voltage to drive the GNA oxidation.

To evaluate the feasibility of a self‐driven, closed‐loop electrochemical system, we connected the GLU‐ and GNA‐fuel cells in series, which successfully illuminated a small light bulb (Figure 4c), demonstrating that electricity can be generated. Thus, the proposed cascade system can facilitate the utilization of GLU for sustainable production of high‐value chemicals while simultaneously generating energy. However, when testing the GLU fuel cell at an output voltage of 0.37 V, it only reaches a maximum GNA yield of 50.49%. This is because the decreasing GLU concentrations results in voltage drop and surface passivation of Au/C catalysts leads to diminishing GLU conversion activity. A fuel cell management system with catalyst regeneration process can in principle enable the anodic oxidation. Nonetheless, to demonstrate the applicability of full GLU oxidation and its tandem conversion to GRA, we here use fixed applied potential at 0.6 and 0.55 V vs RHE for the first‐ and second‐step oxidation, respectively.

To finally assess the overall conversion efficiency and selectivity of GLU‐to‐GRA tandem system, we used the anolyte from GLU oxidation for subsequent oxidation. The products were quantified using HPLC (Figure 4d). The peak at the retention time of ∼6 min corresponds to GNA, which becomes stronger as the electrolysis proceeds. The quantification of GLU conversion and GNA yield at different time is summarized in Figure 4e. In the first‐step oxidation at 0.6 V, the GLU conversion efficiency is nearly 100% and the GNA yield reaches 98% (Figure 4e), which provides a high‐quality solution for the subsequent reaction. In the second‐step oxidation at 0.55 V, the electrolyte containing 98% GNA is oxidized using AuPt/C as the catalyst. HPLC analysis (Figure 4f) shows the decreasing intensity at ∼ 6 min (GNA peak) and the concurrent increasing intensity at ∼16 min (GRA), confirming the conversion of GNA to GRA can be initiated at the low applied potential of 0.55 V. Quantitative analysis reveals a GRA yield of 78% and a GNA conversion rate of 89.7% (Figure 4g). Some minor by‐products such as HCOOH and TA are also detected due to C─C bond cleavage. By cascading these two reactions, the overall conversion rate of GLU is ∼100% and the yield of GRA 76.4%. Notably, our performance is achieved at a much lower overpotential than the literature at >1 V.

We finally present the energy and economic comparison of the one‐step and two‐step tandem processes. First, we evaluate the energy efficiency (EE) of the two systems. We use density functional theory (DFT; See Methods for detail) calculations to estimate the thermodynamic equilibrium potential for Equations (1) and (4), which are 0.32 and 0.15 V, respectively. For one‐step process, the EE_GLU‐to‐GRA_ is 13.8%, assuming an FE_GRA_ of 90% at 1.3 V. In contrast, the EE_GLU‐to‐GNA_ and EE_GNA‐to‐GRA_ for the tandem process is 50% and 24.5%, respectively, assuming an FE_GNA_ of 100% at 0.6 V and FE_GRA_ of 90% at 0.55 V. These values correspond to an EE_GNA‐to‐GRA_ value of 31.8% for the tandem process, substantially higher than that of the one‐step conversion.

**FIGURE 5:**
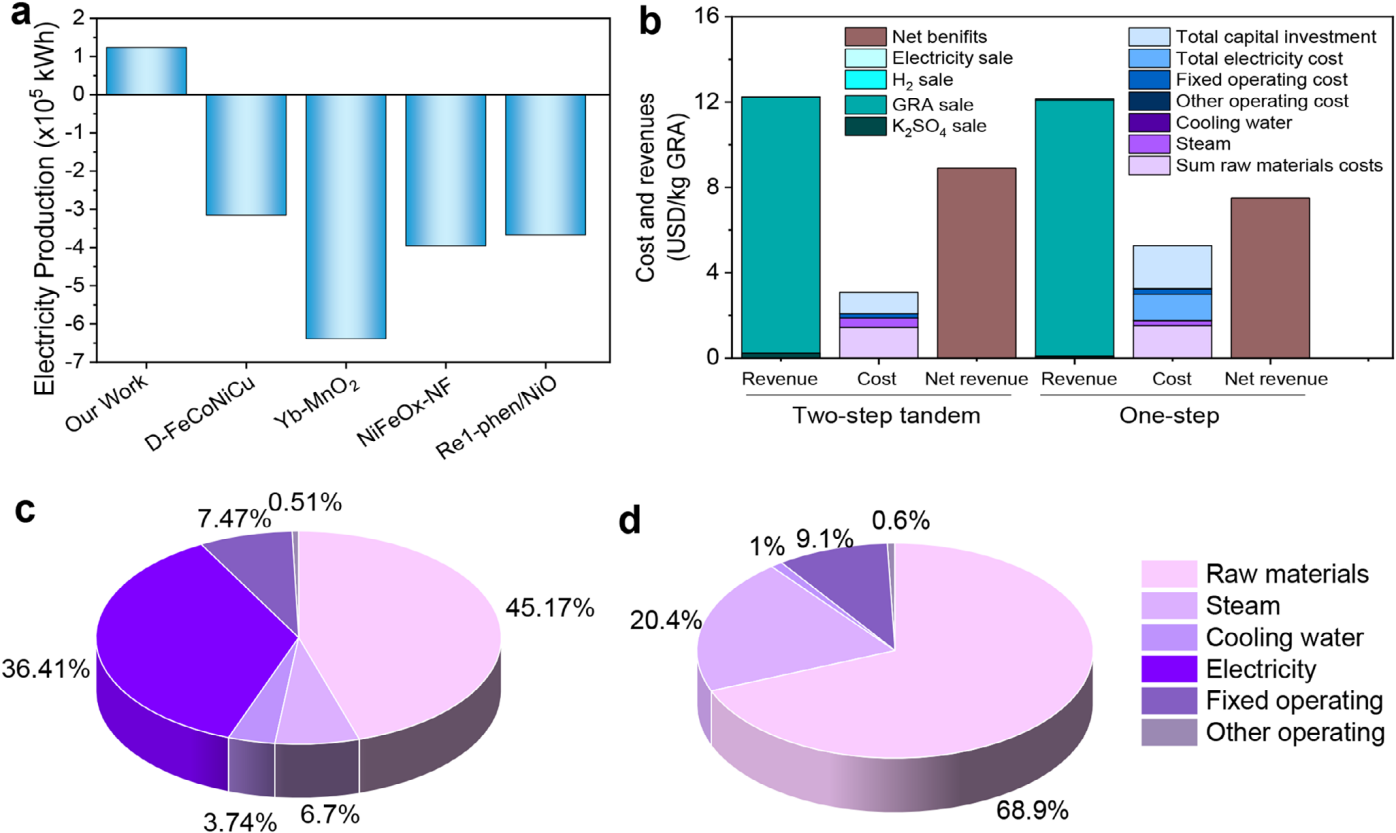
Economic benefits of tandem electrolysis. (a) Comparison of electricity consumption for the production of GRA (1000 tons of GRA per year) by two‐step tandem system GLU oxidation and other literature [26, 44, 45, 46] (coupled with ORR) and (b) Comparison of revenues and costs by two‐step tandem system and one step process. Comparison of the total operating costs of the (c) one‐step process and (d) two‐step tandem system.

To evaluate economic feasibility, we then compared the economic benefit of our system's performance with that of a conventional one‐step electrolyzer. A production scale of 1000 tons of GRA per year was assumed, using the economic and technical parameters outlined in Supplementary Methods 3, along with the flow diagram in Figures S35 and S36. To provide a more comprehensive comparison of energy consumption, we employed two calculation methods. The first method is based on GLU electrooxidation coupled with ORR, while the second method considers the hydrogen evolution reaction (HER) at the cathode and the electrical energy generated by hydrogen. Detailed calculations are provided in Supplementary Methods 2, and the results are summarized in Figures 5a and S37. As shown in Figures 5a and S37, our proposed system offers significant advantages over conventional one‐step electrolyzers, which typically consume substantial amounts of electricity during production. In contrast, our system is projected to generate up to 1.24 × 10^5 ^kWh per 1000 tons of GRA annually, presenting a promising solution for achieving sustainable energy capacity.

Figure 5b illustrates a comparison of the costs and benefits between the two‐step tandem system and one‐step process. Although the total investment cost of the two‐step tandem process is slightly higher than that of the one‐step process, the two‐step tandem process has a higher net revenue to produce 1000 tons of GRA per year, which is mainly attributed to the reduction in the cost of electricity. Figure 5c,d compare the operating cost analysis of the two GLU oxidation routes. The main components are raw material, utility, and fixed operating costs, with raw material costs being the largest component. The cost of electricity accounts for 0% of the total operating cost for tandem system, while in the single‐step GLU electrolysis system, the cost of electricity represents 36.41%. Thus, by operating electrolysis at minimum energy consumption, the tandem process can substantially reduce the production cost. Revenue streams include GRA, potassium sulfate (K_2_SO_4_), electricity sale and hydrogen (H_2_), with GRA being the primary revenue source. The current market price of GRA is US $12 kg^−1^. After calculating capital expenditures, operating expenditures, and revenues, our two‐step tandem system generated net revenues of $8.90 million, which is more than the profitability of the conventional one‐step process (Figure 5b), making it highly competitive in the market. Besides, we conducted a comparison of the net revenue generated from producing 1,000 tons of GRA using various published catalysts. As illustrated by Figure S38, after deducting total raw material, utility, and operating costs, our tandem two‐step system demonstrates significantly higher net revenue compared to other one‐step processes, highlighting its economic benefits. The findings underscore the promise of this innovative approach for advancing green chemistry and energy efficiency in chemical production.

## 3 Conclusion

In conclusion, our study proposed a novel two‐step tandem system for the conversion of GLU to GNA and subsequently to GRA at low overpotentials. We identified Au/C and AuPt/C as optimal catalysts for the first and second oxidation steps, respectively, demonstrating efficient conversion rates at low applied potentials: GLU to GNA at 0.6 V and GNA to GRA at 0.55 V, substantially lower than the reported applied potentials exceeding 1.2 V. Since these potentials are lower than the oxygen reduction reaction, the construction of a fuel cell can potentially convert GLU to GRA while generating electricity, forming a closed‐loop electrochemical system with an estimated electricity generation of 1.24 × 10^5^ kWh per 1 000 tons of GRA produced. In stark contrast to the conventional one‐step process, an energy consumption of 4.31 × 10^5^ kWh is required to produce 1,000 tons of GRA. We estimate that our tandem two‐step system achieves a net revenue of $8.90 million, surpassing that of the one‐step process, highlighting its economic advantages.

Our study demonstrated that by dividing a one‐step reaction into two steps, the tandem system improves product selectivity, chemical conversion, and reduces energy consumption. This two‐step tandem process can also be applied to other applications such as C‐N coupling and CO_2_ reduction [47, 48]. These reactions also involve sequential reactions, where tandem electrolysis provides opportunities to optimize each step with minimal energy consumption. Currently, the current density at low potential is lower than those reported at high potential. Future catalyst development in improving current densities at low potentials will make the tandem process more compelling for biomass valorization. Overall, this innovative approach not only enhances the sustainable utilization of biomass for high‐value chemical production but also offers a promising pathway for energy conservation in green chemistry.

## 4 Materials and Methods

Carbon powder Vulcan XC‐72R (GP‐3919, Cabot) was used as support. K_2_PtCl_4_ (Pt 47%) and HAuCl_4_ (Au 49%) (CDH, India) were used as platinum and gold precursors, respectively. NaBH4 (CDH, India) was used as a reducing agent. All aqueous solutions were prepared using ultrapure water (18.2 MΩ cm Millipore‐MilliQ) during catalyst preparation. Nafion dispersion (DE‐521, DuPont, USA) and acetone (>99.5% purity) (Merck) were used for catalyst ink preparation. Catalyst‐coated carbon paper (Toray 060) was used as the working electrode. D‐glucose (>99% pure, Macklin), D‐gluconic acid (>98% pure, Macklin), D‐glucaric acid (>98% pure, Macklin) were used for testing. KOH (>85% assay, flakes purified) (Merck) was used as electrolyte. Copper foam and Copper(I) oxide (>99% pure, Macklin) were used as reference experiments.

### 4.1 Synthesis of the Au/C and AuPt/C Catalysts

First, an aqueous solution of 100 mg/l HAuCl_4_ was prepared, and glucose was added as a protective agent (metal: glucose 1:50 w/w). A freshly prepared 0.1 M NaBH_4_ solution was dropwise added to the metal dispersion (metal: NaBH_4_ 1:1 w/w) under vigorous stirring conditions, and a black colloidal dispersion was formed. Carbon (Vulcan XC‐72R) was added to the metal dispersion under stirring to immobilize the metal particles. The slurry was left overnight and filtered when the solution became clear. The solid obtained was washed several times with distilled water and air‐dried for 12 h. For the AuPt/C catalyst (metal ratio 1:1), the synthesis method is the same as Au/C, except that an equal amount of K_2_PtCl_4_ is added during the synthesis of Au/C.

### 4.2 Synthesis of NiFe/NF Catalysts

NiFe/NF was synthesized referring to Zhao et al., with some adjustments [45]. First, the NF was pretreated by sonication in 5 M HCl solution for 20 min to remove the nickel oxide layer on the surface, followed by rinsing with water and ethanol, and then dried in air. Electrodeposition was carried out in a standard three‐electrode electrochemical cell, with the working, counter, and reference electrodes as NF, a platinum sheet, and Ag/AgCl (3 M KCl), respectively. The electrolytes were 3 mM Ni(NO_3_)_2_·6H_2_O and 3 mM Fe(NO_3_)·9H_2_O, and electrodeposition was carried out at constant potential for 300 s at −1.0 V (versus Ag/AgCl) to obtain the NiFe/NF. After deposition, the Ni─Fe alloy was separated from the electrolyte and washed with water and ethanol sequentially, then sonicated in ethanol for a few seconds and dried naturally.

### 4.3 Preparation of Working Electrodes

To obtain a homogeneous ink, 5 mg of catalyst, 950 mL of ethanol, and 50 µL of Nafion binder solution (Sigma–Aldrich, Nafion 117, 5 wt.%) were mixed, followed by sonication for 30 min until the ink was uniformly dispersed. Then, 100 µL of the homogeneously dispersed ink was dip‐coated onto 1 × 1 cm^2^ carbon paper and after drying naturally to obtain the desired catalyst loading (0.5 mg cm^−2^).

### 4.4 Electrochemical Measurements

The Electrochemical measurements were conducted with a CHI 760D electrochemical workstation (ChenHua Instrument Inc., China), in which the Ag/AgCl electrode and Pt plate were used as the reference electrode and the counter electrode, the pre‐prepared Au/C or AuPt/C coated on carbon paper was used as the working electrode in the first or second step, respectively. Passivation occurs during the use of the working electrode. When the current is observed to decrease to a threshold of 0 mA due to passivation of the noble metal, we reactivate the electrode by taking 50‐cycle cyclic voltammetry between 0.2 and 0.8 V to restore its activity. The potentials vs. Ag/AgCl measured in the experiments were converted to potentials vs. Reversible Hydrogen Electrode (RHE) according to the Nernst equation:

$$E\left(\mathrm{RHE}\right)=E\left(\mathrm{Ag}/\mathrm{AgCl}\right)+0.059\;\times \;\mathrm{pH}+0.197$$

The anodic and cathodic electrolytes were 1 M KOH + 0.01 M GLU or GNA and 1 M KOH, respectively, separated by an alkaline membrane (FAA‐3‐PK‐75, Fumatech). The volume of cathode and anode solution is 10 mL.

### 4.5 Products Analysis and Faradaic Efficiencies (FE), Selectivity Calculation

GLU concentration: The products of GLU oxidation were analyzed using ^1^H NMR spectroscopy with solvent (H_2_O) inhibition. Specifically, 450 µL of electrolytes were mixed with 50 µL of 10 mM dimethylsulfoxide in D_2_O as an internal standard for ^1^H NMR analysis.

GNA and GRA concentrations: Concentration changes of GNA and GRA during electrolysis were monitored by HPLC equipped with ultraviolet‐visible detector. The mobile phases were dipotassium hydrogen phosphate and dipotassium hydrogen phosphate and tetrabutylammonium hydrogensulphate solution with a constant flow rate of 0.7 mL min^−1^. For each sample, 0.5 mL of electrolyte solution was drawn from the reaction cell and neutralized with 2.0 m H_2_SO_4_. Then 10 µL of the neutralized electrolyte solution was injected directly onto a Kromasil C18 column at 35°C.

The conversion of GLU (*η*_glu_) and its yield of oxidation products (*Y_p_*) were calculated as follows Equations (7) and (8):

(7) ηglu=(1−Cglu/C0−glu)×100%

(8) Yp=Cp/C0−glu×100%

where *C*_0‐glu_ and *C*_glu_ are the original GLU concentration and GLU concentration at different reaction times, respectively, and *Cp* is the concentration of GLU oxidation products (GNA and GRA) at different reaction times.

The Faradaic efficiency (*FE*) was calculated with the following Equation (9):

(9) $$\mathrm{FE}=\frac{n_{\mathrm{certain}\;\mathrm{product}}\times Z\times F}{Q}\times 100\%$$

where *n* is the total amount of a certain product produced (in moles), *z* is the number of electrons transferred to correspondingly produce 1 mole of products, which is 2 for GLU to GNA, 6 for GLU to GRA, 4 for GNA to GRA; *F* is the Faraday constant of 96 485 C mol^−1^; *Q* is the charge passing through the circuit during electrolysis. Q = J × S × t, where J (A cm^−2^) is the current density at a specific applied potential, S is the electrode area (cm^2^), and *t* is the reaction time (seconds).

The energy efficiency (*EE*) was calculated with the following Equation (10):

(10) $$EE=\frac{n\times F\times E_{eq}}{n\times F\times E_{appl}}\times FE$$

where *n* is the number of electrons, *E_eq_* is the thermodynamic equilibrium potential. Using DFT calculations, the *E_eq_* for Equations (1) and (4) are calculated to be 0.32 and 0.15 V, respectively; DFT calculations have been carried out with the DMol3 package embedded in Material Studio. The geometrical optimization has adopted the GGA functionals with BLYP. All the optimized structures were confirmed as potential minima. The calculations of the theoretical potential are based on the Nernst equation as ΔG = ‐nFE at 298.15 K for each reaction process, where the zero‐point vibration energy (ZPVE) has been included in the calculations of ΔG. The Convergence thresholds are established as 2 × 10^−5^ eV per atom for total energy and 0.005 Å for atomic displacement during the optimization procedures. *E_appl_* is the actual applied potential.

## Conflicts of Interest

The authors declare no conflicts of interest.

## Supporting information




**Supporting File**: adma71931‐sup‐0001‐SuppMat.docx

## Data Availability

The data that support the findings of this study are available from the corresponding author upon reasonable request.
