# Supplementary material for: Energy‐Efficient, Sustainable Cascade Glucose Electrooxidation into Glucaric Acid
Source: Adv Mater. 2025 Dec 24;38(10):e19531. doi: 10.1002/adma.202519531 (PMC12910541; doi:10.1002/adma.202519531)
Supplement: Supplementary file 1 — Supporting File: adma71931‐sup‐0001‐SuppMat.docx [file ADMA-38-e19531-s001.pdf]

Supporting Information

**Supplementary Text**

Supplementary Methods 1. Characterizations of the catalysts

The morphology of the samples was characterized using a scanning electron microscope (SEM, TESCAN MIRA LMS, Czech Republic) and a transmission electron microscope (TEM, JEOL JEM-F200, Japan) with an energy dispersive X-ray spectroscopy (EDS) equipped with a Thermo-Fisher EDS detector at 15 kV. XRD patterns were obtained with a Smart Lab diffractometer. X-ray photoelectron spectroscopy (XPS) analysis was performed via an X-ray photoelectron spectrometer (Thermo Scientific K-Alpha, UK) using monochromatized Al Kα radiation (1486.6 eV). The binding energy was corrected by the C 1s peak at 284.8 eV. The stability of the catalyst was measured using an ICP-OES Optima 8000 spectrometer to measure the solution after the reaction. The samples were digested overnight in aqua regia (concentrated hydrochloric acid and concentrated nitric acid in a 3:1 volume ratio) and diluted to the desired concentration.

Supplementary Methods 2. Calculation of the electricity power generation

A production rate of 1000 tons per year of glucaric acid (GRA) is assumed, and every mole of glucose (GLU) consumed generates 0.76 moles of GRA, and only the electricity generated in the first step is considered.

The molar mass of GRA is 0.21 kg/mol, the amount of substance of 1000 tons of GRA is $n_{GRA}= 4.75\times10^6$ mol. Given that the yield of GRA is 76.4%, the amount of GLU required is $n_{GLU} = 6.23\times10^6$ mol. One molecule of GLU loses 2 electrons when converted to gluconic acid (GNA). So the amount of substance of transferred electrons is $n_e=2\times n_{GLU}=1.24\times10^7$ mol. According to Faraday's constant F = 96485 C/mol, the charge $Q = n_e\times F = 1.20\times10^{12}$ C. Assuming the electrode area is S $cm^2$, the power $P = 8.9\times10^{-4}S$ W. According to P = UI (U = 0.37 V), the current $I = P/U = 2.4\times10^{-3}$ *SA*. From I = Q/t, the reaction time $t = 5.0\times10^{14}/S$ s. According to W = UIt (U = 0.37 V), $W = 0.37V\times2.40\times10^{-3}SA\times5.0\times10^{14}/S = 4.47\times10^{11}$ J, Since 1 kW/h = $3.60\times10^6$ J, thus the electrical energy generated in the first step (conversion of GLU to GNA) is

$W = 1.24\times10^5$ kW/h.

For calculating the electricity power consumed for the one-step process, we performed two calculations methods of the one-step process for a more comprehensive comparison. The first method is based on GLU

electro-oxidation coupled with the oxygen reduction reaction (ORR), while the second method considers the hydrogen evolution reaction (HER) at the cathode and the electrical energy generated by hydrogen.

**The first method:**

Usually, the voltage for the electro-oxidation of GLU to GRA is 1.30 V, and the cathode undergoes oxygen reduction at a potential of 0.85 V, so that the potential to be applied is 0.45 V. Likewise, a production rate of 1000 tons per year of GRA is assumed. Each mole of GRA formation from GLU involves a 6-electron transfer. The theoretical total charge is: $Q_{theoretical} = nF = 4.75\times10^{6}$ mol×6×96485 C/mol = $2.76\times10^{12}$ C. With an 80% yield of GRA, the actual required charge is $Q_{actual} = Q_{theoretical}/0.8 = 2.76\times10^{12}$ C/0.8 = $3.45\times10^{12}$ C. W = Q×V = $3.45\times10^{12}$ C×0.45 V = $1.55\times10^{12}$ J = $4.31\times10^{5}$ kWh.

For Yb-$MnO_2$ catalyst, the voltage for the electro-oxidation of GLU to GRA is 1.558 V, and the cathode undergoes oxygen reduction at a potential of 0.85 V, so that the potential to be applied is 0.708 V. Likewise, a production rate of 1000 tons per year of GRA is assumed. Each mole of GRA formation from GLU involves a 6-electron transfer. The theoretical total charge is: $Q_{theoretical} = nF = 4.75\times10^{6}$ mol×6×96485 C/mol = $2.76\times10^{12}$ C. With an 84.6% yield of GRA, the actual required charge is $Q_{actual} = Q_{theoretical}/0.846 = 2.76\times10^{12}$ C/0.846 = $3.26\times10^{12}$ C. W = Q×V = $3.26\times10^{12}$ C×0.708 V = $6.39\times10^{5}$ kWh.

For NiFeOx-NF//NiFeNx-NF catalyst, the voltage for the electro-oxidation of GLU to GRA is 1.3 V, and the cathode undergoes oxygen reduction at a potential of 0.85 V, so that the potential to be applied is 0.45 V. Likewise, a production rate of 1000 tons per year of GRA is assumed. Each mole of GRA formation from GLU involves a 6-electron transfer. The theoretical total charge is: $Q_{theoretical} = nF = 4.75\times10^{6}$ mol×6×96485 C/mol = $2.76\times10^{12}$ C. With an 87% yield of GRA, the actual required charge is $Q_{actual} = Q_{theoretical}/0.87 = 2.76\times10^{12}$ C/0.87 = $3.17\times10^{12}$ C. W = Q×V = $3.17\times10^{12}$ C×0.45 V = $3.96\times10^{5}$ kWh.

For D-FeCoNiCu-LDH/NF catalyst, the voltage for the electro-oxidation of GLU to GRA is 1.22 V, and the cathode undergoes oxygen reduction at a potential of 0.85 V, so that the potential to be applied is 0.37 V. Likewise, a production rate of 1000 tons per year of GRA is assumed. Each mole of GRA formation from GLU involves a 6-electron transfer. The theoretical total charge is: $Q_{theoretical} = nF = 4.75\times10^{6}$ mol×6×96485 C/mol = $2.76\times10^{12}$ C. With an 90% yield of GRA, the actual required charge is $Q_{actual} = Q_{theoretical}/0.9 = 2.76\times10^{12}$ C/0.9 = $3.06\times10^{12}$ C. W = Q×V = $3.06\times10^{12}$ C×0.37 V = $3.15\times10^{5}$ kWh.

For Re1-phen/NiO catalyst, the voltage for the electro-oxidation of GLU to GRA is 1.30 V, and the cathode undergoes oxygen reduction at a potential of 0.85 V, so that the potential to be applied is 0.45 V. Likewise, a production rate of 1000 tons per year of GRA is assumed. Each mole of GRA formation from GLU involves a 6-electron transfer. The theoretical total charge is: $Q_{theoretical} = nF = 4.75\times10^{6}$

mol×6×96485 C/mol = 2.76×$10^{12}$ C. With an 94% yield of GRA, the actual required charge is $Q_{actual}$ = $Q_{theoretical}$/0.94 = 2.76×$10^{12}$ C/0.94 = 2.93×$10^{12}$ C. W = Q×V = 2.93×$10^{12}$ C×0.45 V = 3.67×$10^5$ kWh. Overall, our system can generate 4.31×$10^5$ kWh of electricity, while for the one-step system, Yb-$MnO_2$, NiFeOx-NF//NiFeNx-NF, D-FeCoNiCu-LDH/NF, and Re1-phen/NiO catalysts, the electrical energy consumed is 6.39×$10^5$ kWh, 3.96×$10^5$ kWh, 3.15×$10^5$ kWh, and 3.67×$10^5$ kWh, respectively.

**The second method:**

For Yb-$MnO_2$ catalyst, the total transferred electrons = 4.75×$10^6$ mol × 6 /0.846 = 3.3×$10^7$ mol $e^-$, the actual required charge is $Q_{actual}$ = 3.3×$10^7$ mol $e^-$×96485 C/mol = 3.25×$10^{12}$ C. Total Electricity Consumption is 3.25 ×$10^{12}$ C × 1.558 V = 5.06 ×$10^{12}$ J = 14.06 ×$10^5$ kWh. The $H_2$ production in cathodic is: $H_2$ moles = 3.33×$10^7$ mol $e^-$ / 2 = 1.66×$10^7$ mol, $H_2$ mass = 1.66×$10^7$ mol × 0.002016 kg/mol = 33465.6 kg. The electricity formed from $H_2$ is: $H_2$ chemical energy = 33465.6 kg × 120×$10^6$ J/kg = 4.01×$10^{12}$ J. Assuming the fuel cell efficiency is 60%, so the recoverable electricity is = 4.01×$10^{12}$ J × 60%= 6.69 ×$10^5$ kWh. The net electricity consumption is: 14.06 ×$10^5$ kWh - 6.69 ×$10^5$ kWh = 7.37 ×$10^5$ kWh.

For NiFeOx-NF//NiFeNx-NF catalyst, the total transferred electrons = 4.75×$10^6$ mol × 6 /0.87 = 3.27×$10^7$ mol $e^-$, the actual required charge is $Q_{actual}$ = 3.27×$10^7$ mol $e^-$×96485 C/mol = 3.16 ×$10^{12}$ C. Total Electricity Consumption is 3.16 ×$10^{12}$ C × 1.3 V = 4.11×$10^{12}$ J = 11.41×$10^5$ kWh. The $H_2$ production in cathodic is: $H_2$ moles = 3.27×$10^7$ mol $e^-$ / 2 = 1.63×$10^7$ mol, $H_2$ mass = 1.63×$10^7$ mol × 0.002016 kg/mol = 32961.6 kg. The electricity formed from $H_2$ is: $H_2$ chemical energy = 32961.6 kg × 120×$10^6$ J/kg = 3.96×$10^{12}$ J. Assuming the fuel cell efficiency is 60%, so the recoverable electricity is = 3.96×$10^{12}$ J × 60%= 6.59×$10^5$ kWh. The net electricity consumption is: 11.41 ×$10^5$ kWh - 6.59 ×$10^5$ kWh = 4.82 ×$10^5$ kWh.

For D-FeCoNiCu-LDH/NF catalyst, the total transferred electrons = 4.75×$10^6$ mol × 6 /0.9 = 3.16×$10^7$ mol $e^-$, the actual required charge is $Q_{actual}$ = 3.16×$10^7$ mol $e^-$×96485 C/mol = 3.06×$10^{12}$ C. Total Electricity Consumption is 3.06 ×$10^{12}$ C × 1.220 V = 3.73×$10^{12}$ J = 10.3 ×$10^5$ kWh. The $H_2$ production in cathodic is: $H_2$ moles = 3.16×$10^7$ mol $e^-$ / 2 = 1.58×$10^7$ mol, $H_2$ mass = 1.58×$10^7$ mol × 0.002016 kg/mol = 31920 kg. The electricity formed from $H_2$ is: $H_2$ chemical energy = 31920 kg × 120×$10^6$ J/kg = 3.83×$10^{12}$ J. Assuming the fuel cell efficiency is 60%, so the recoverable electricity is = 3.83×$10^{12}$ J × 60%= 6.38 ×$10^5$ kWh. The net electricity consumption is: 10.3 ×$10^5$ kWh - 6.38 ×$10^5$ kWh = 3.92 ×$10^5$ kWh.

For Re1-phen/NiO catalyst, the total transferred electrons = 4.75×$10^6$ mol × 6 /0.94 = 3.03×$10^7$ mol $e^-$, the actual required charge is $Q_{actual}$ = 3.03×$10^7$ mol $e^-$×96485 C/mol = 2.92×$10^{12}$ C. Total Electricity Consumption is 2.92×$10^{12}$ C × 1.3 V = 3.79×$10^{12}$ J = 9.54×$10^5$ kWh. The $H_2$ production in cathodic is: $H_2$

moles = $3.03\times10^7$ mol $e^-$ / 2 = $1.52\times10^7$ mol, $H_2$ mass = $1.52\times10^7$ mol × 0.002016 kg/mol = 30643.2kg. The electricity formed from $H_2$ is: $H_2$ chemical energy = 30643.2 kg × $120\times10^6$ J/kg = $3.67\times10^{12}$ J. Assuming the fuel cell efficiency is 60%, so the recoverable electricity is = $3.67\times10^{12}$ J × 60%= 6.12 $\times10^5$ kWh. The net electricity consumption is: 9.54 $\times10^5$ kWh - 6.12 $\times10^5$ kWh = 3.42 $\times10^5$ kWh.

In summary, when considering the electricity generated from hydrogen, Yb-$MnO_2$, NiFeOx-NF//NiFeNx-NF, D-FeCoNiCu-LDH/NF, and Re1-phen/NiO catalysts, the electrical energy consumed is 7.37 $\times10^5$ kWh, 4.82 $\times10^5$ kWh, $3.92\times10^5$ kWh, and $3.42\times10^5$ kWh, respectively.

Supplementary Methods 3. Calculation of the cost and benefit for the GLU electrolysis

To simplify the calculations, we assume that the total annual production of GRA is 1000 tons. The conversion of GLU is ~100% and the yield of GRA is 76.4%. As GNA is an intermediate product, its economic value and separation costs are not considered. At a fixed production rate of 1,000 tons of GRA per year, GLU demand is estimated at 1122 tons annually. Using a GLU concentration of 0.01 M and a KOH concentration of 1.0 M, the water and KOH requirement are 678000 tons and 381 tons per year, respectively. Electrochemical oxidation of GLU under alkaline conditions produces the potassium salt products, potassium gluconate ($C_6H_{11}KO_7$) and potassium gluconate ($C_6H_8K_2O_8$). Therefore, before recycling the GRA, the potassium salts in the liquid mixture are neutralised with $H_2SO_4$ (3324 tons per year at 10%) to produce $K_2SO_4$ (595 tons per year), which is a valuable by-product of fertiliser production. The neutralized mixture is then processed in a flash drum distillation column to remove solvent (water), and the remaining concentrate is sent to a crystallizer for the recovery of target chemicals (GRA and $K_2SO_4$). The solid mixture is subsequently isolated and purified through two-stage recrystallization. A flow schematic of this process is presented in Figure S34.

For the conventional one-step electrocatalytic oxidation of GLU, similarly, a total production of 1000 tons per year of GRA was assumed, with a GLU conversion rate of 90% and the selectivity of GRA is 80%. This results in an estimated GLU consumption of approximately 1200 tons per year, yielding 230 tons of GNA at the anode and 31 tons of hydrogen at the cathode. The KOH in the liquid mixture is neutralised with $H_2SO_4$ (10 wt.%, 3550 tons per year) prior to recovery of GRA. The neutralised mixture is then transferred to a distillation column to remove the solvent and the remaining concentrated liquid is fed to a crystalliser to recover the target chemicals (GRA and $K_2SO_4$). Subsequently, the solid mixture is obtained by separation and purification via two-stage recrystallisation. A flow diagram of the above process is shown in Figure S35.

Based on the above discussion, the economic benefits of D-FeCoNiCu-LDH/NF are calculated as follows: A total production of 1000 tons per year of GRA was assumed, with a GLU conversion rate of 90% and the selectivity of GRA is 90%. This results in an estimated GLU consumption of approximately 1058.4 tons per year, yielding 35.27 tons of hydrogen at the cathode. The amounts of KOH and 10 wt.% $H_2SO_4$ required are 329.28 tons and 2881.2 tons respectively, and the mass of $K_2SO_4$ produced is 511.46 tons. The unit price of each product is listed in Table S2, and the net profit margin calculated based on Table S6 is 6.00 million dollars.

For Re1-phen/NiO catalyst, a total production of 1000 tons per year of GRA was assumed, with a GLU conversion rate of 90% and the selectivity of GRA is 94%. This results in an estimated GLU consumption of approximately 1013.4 tons per year, yielding 33.77 tons of hydrogen at the cathode. The amounts of KOH and 10 wt.% $H_2SO_4$ required are 315.28 tons and 2758.7 tons respectively, and the mass of $K_2SO_4$ produced is 489.7 tons. The unit price of each product is listed in Table S2, and the net profit margin calculated based on Table S6 is 6.03 million dollars.

For Yb-$MnO_2$ catalyst, a total production of 1000 tons per year of GRA was assumed, with a GLU conversion rate of 90% and the selectivity of GRA is 84.6%. This results in an estimated GLU consumption of approximately 1125 tons per year, yielding 37.52 tons of hydrogen at the cathode. The amounts of KOH and 10 wt.% $H_2SO_4$ required are 350 tons and 3062.5 tons respectively, and the mass of $K_2SO_4$ produced is 544.11tons. The unit price of each product is listed in Table S2, and the net profit margin calculated based on Table S6 is 5.94 million dollars.

For NiFe$O_x$-NF//NiFe$N_x$-NF catalyst, a total production of 1000 tons per year of GRA was assumed, with a GLU conversion rate of 90% and the selectivity of GRA is 87%. This results in an estimated GLU consumption of approximately 1094.4 tons per year, yielding 36.49 tons of hydrogen at the cathode. The amounts of KOH and 10 wt.% $H_2SO_4$ required are 340.48 tons and 2929.2 tons respectively, and the mass of $K_2SO_4$ produced is 544.11tons. The unit price of each product is listed in Table S2, and the net profit margin calculated based on Table S6 is 5.97 million dollars.

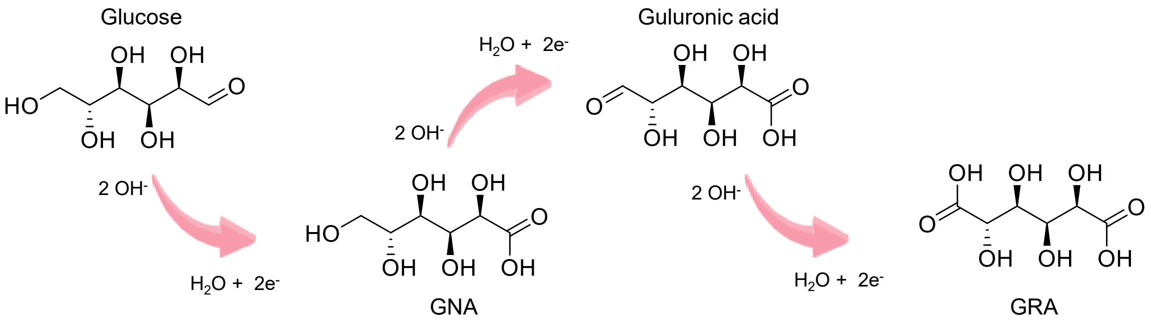


**Figure S1.** Schematic illustration of the possible pathway for the electrochemical oxidation of GLU to GNA and GRA.

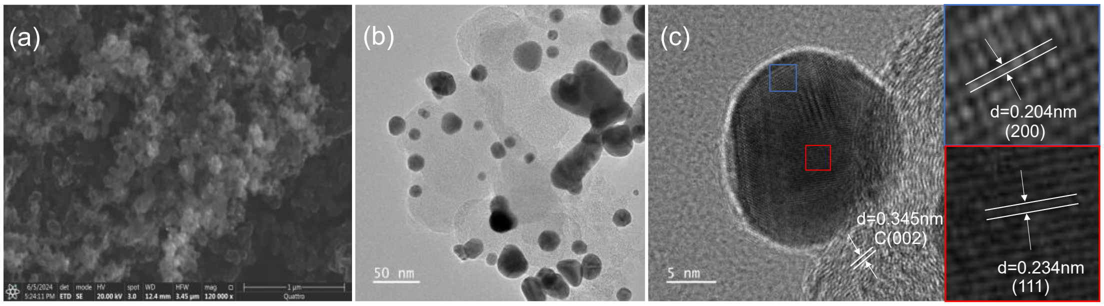


**Figure S2.** (a) SEM, (b) TEM and (c) HRTEM pattern of the Au/C.

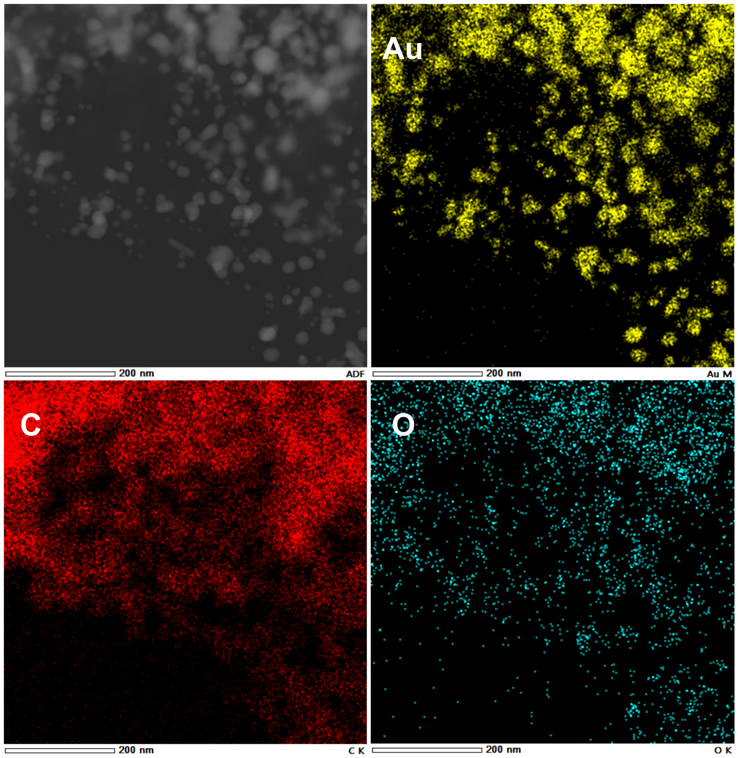


**Figure S3.** SEM-EDS elemental mapping of the Au/C electrode catalyst.

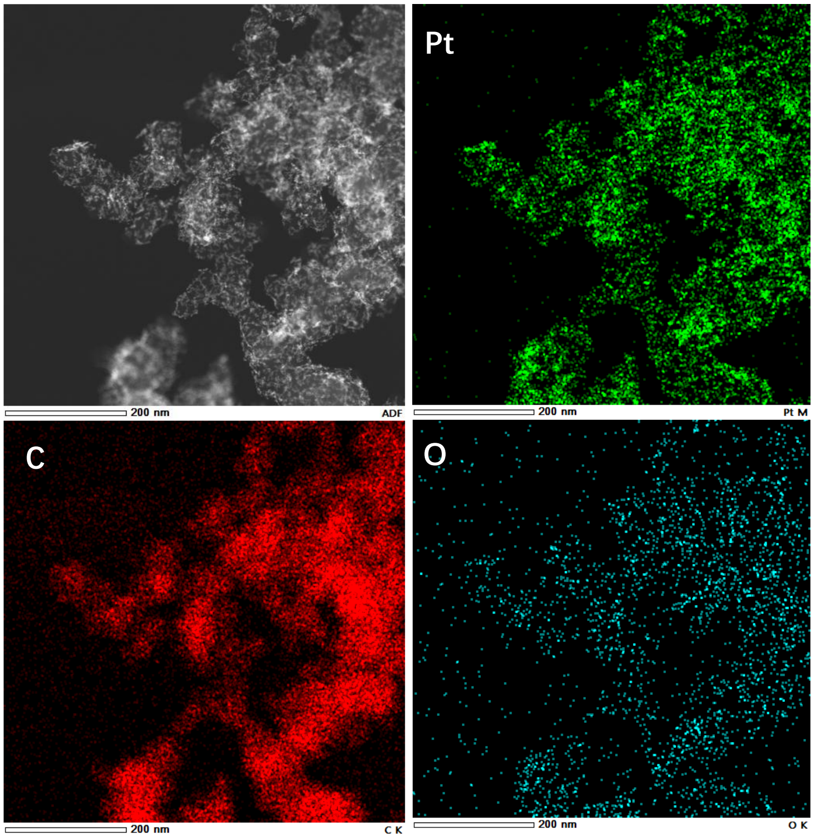


**Figure S4.** SEM-EDS elemental mapping of the Pt/C electrode catalyst.

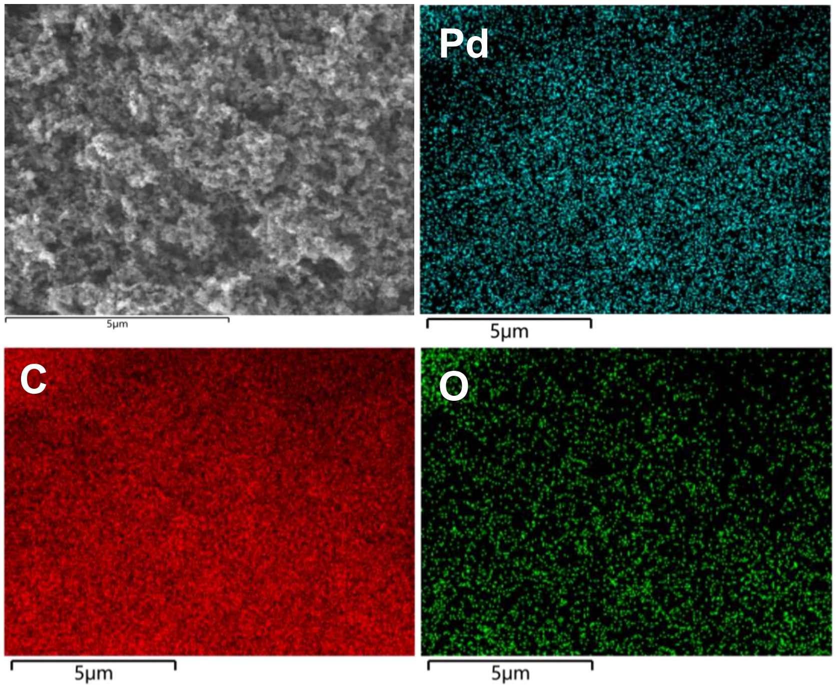


**Figure S5.** SEM-EDS elemental mapping of the Pd/C electrode catalyst.

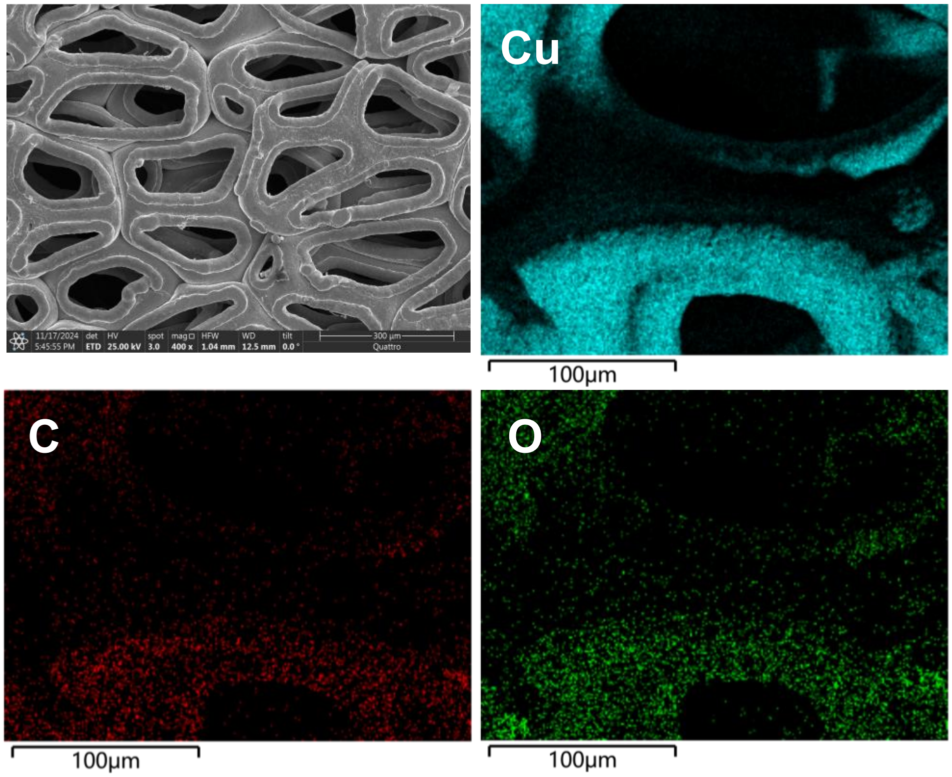


**Figure S6.** SEM-EDS elemental mapping of the Cu Foam electrode catalyst.

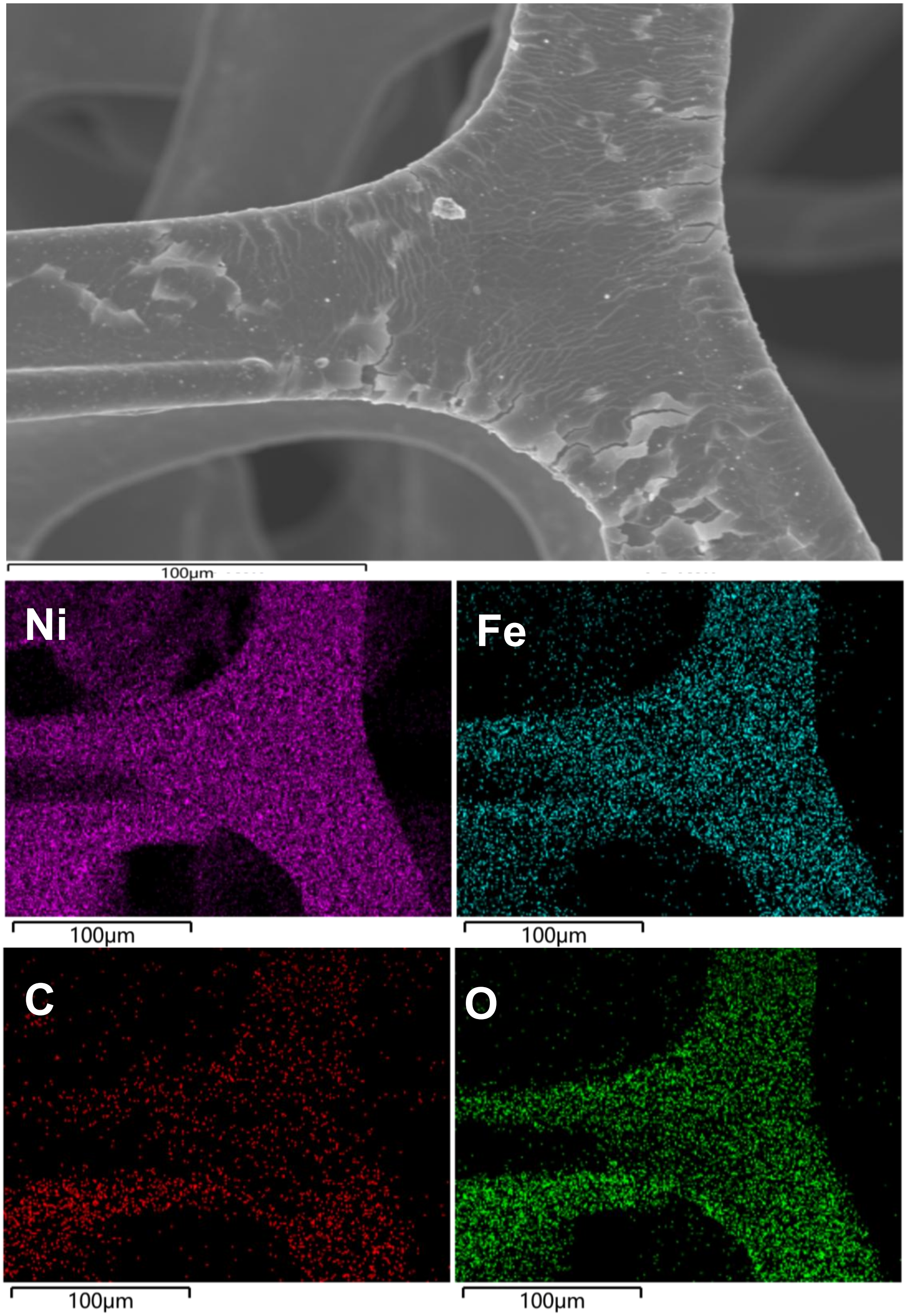


**Figure S7.** SEM-EDS elemental mapping of the NiFe electrode catalyst.

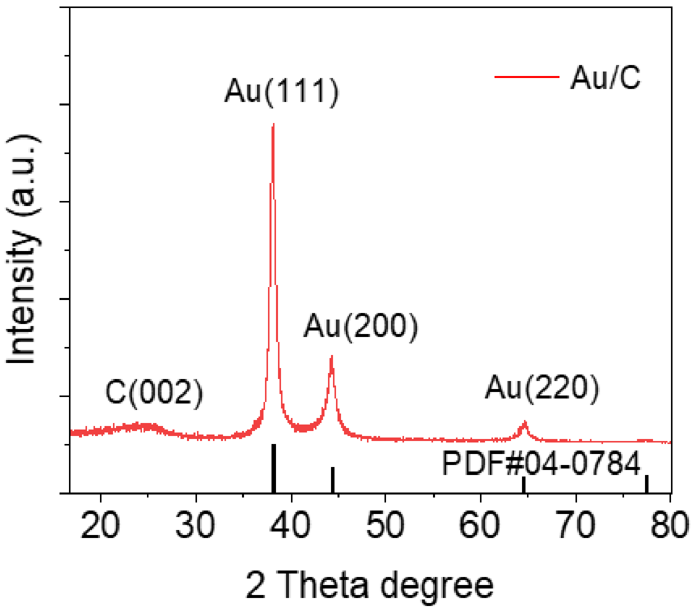


**Figure S8.** XRD pattern of the Au/C.

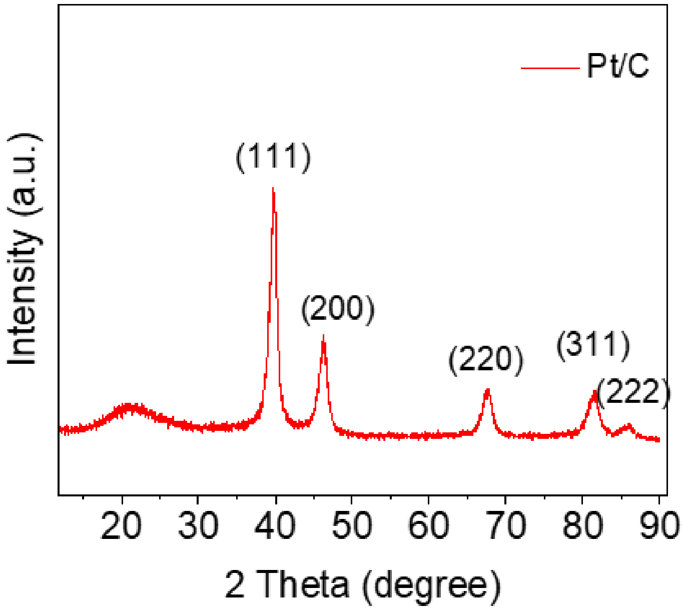


**Figure S9.** XRD curves of Pt/C. The peaks appeared at a 2θ of 39.58°, 46.04°, 67.09°, and 87° represented the characteristic diffraction peaks of Pt (111), Pt (200), Pt (311), and, Pt (222), respectively. These peaks are attributed to the face-centered cubic (fcc) phase structure of Pt. A broad peak at about 2θ = 25° assigned to graphitic carbon (002).

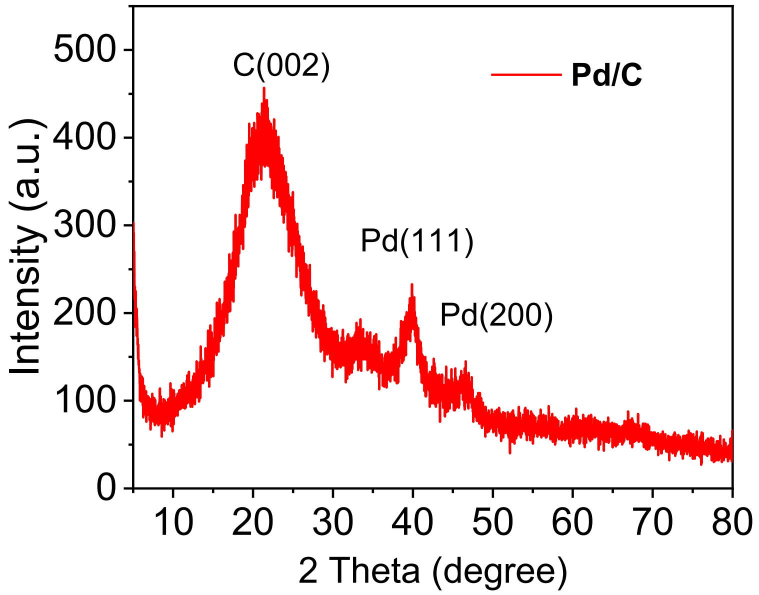


**Figure S10.** XRD curves of Pd/C. The X-ray diffraction (XRD) spectra of the Pd/C catalysts reveal structural characteristics of both carbon and palladium. The diffraction peak at a 2θ angle of approximately 25.0° corresponds to the graphitic structure of carbon, specifically the (002) plane. Additionally, the peaks observed at 2θ angles of 39.6°, 44.8°, 67.5°, and 81.6° are attributed to the face-centered cubic (fcc) crystal structure of palladium, confirming its metallic state on the carbon support.

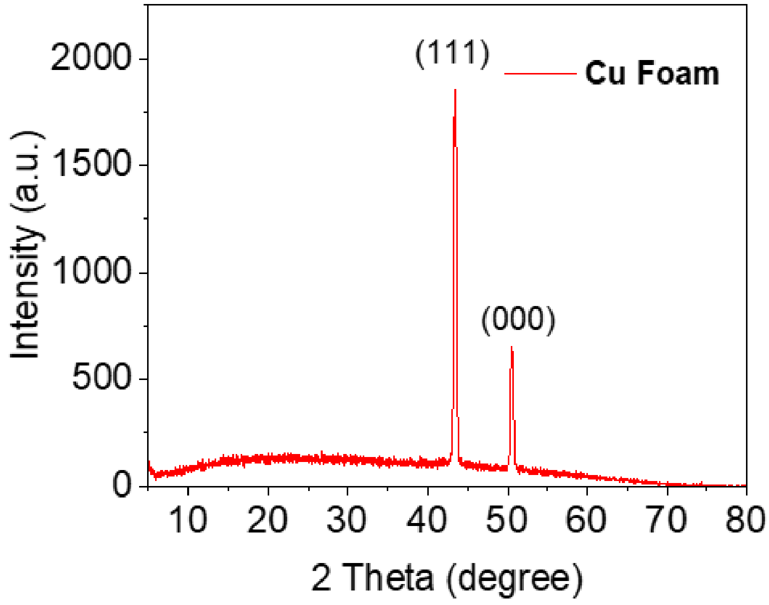


**Figure S11.** XRD curves of Cu Foam. There are two strong reflection peaks at 2θ = 43.58, 50.51°, which can indicate the (111) and (200), crystal planes of Cubic Cu structure (JCPDS No. 02-1225).

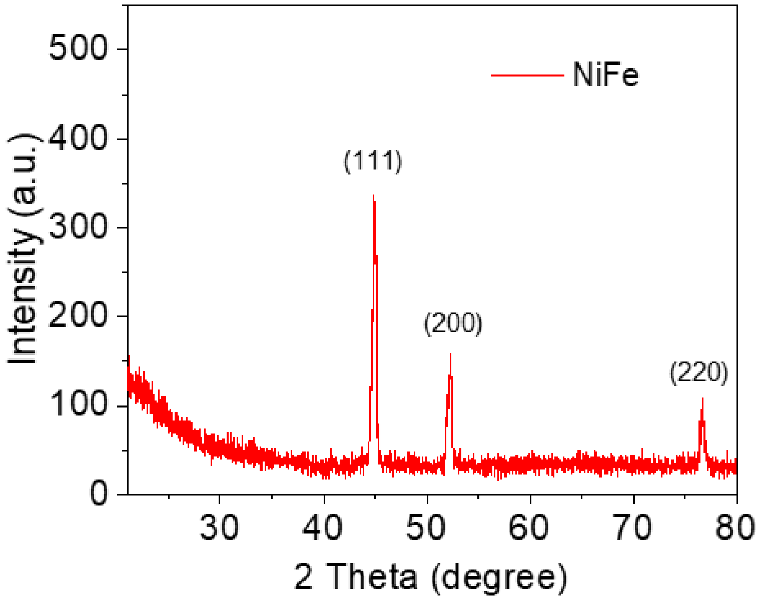


**Figure S12.** XRD curves of NiFe/NF. The XRD pattern exhibited three diffraction peaks of the NF at 44.5°, 51.8° and 76.4°, respectively, confirming that the NiFe deposited onto NF is amorphous in nature. This result is in agreement with the literature and confirms the successful synthesis of NiFe/NF .[1]

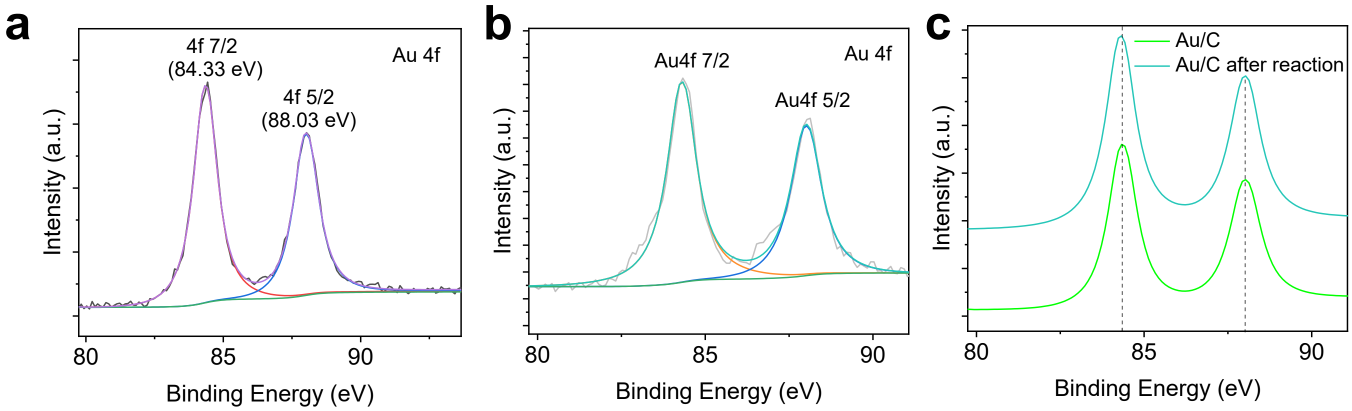


**Figure S13.** XPS pattern of the (a) fresh Au/C, (b) after reaction, and (c) comparison of fitted Au/C before and after the GLU electrooxidation reaction. The deconvoluted XPS peaks at 84.33 and 88.03 eV are attributed to the 4f 7/2 and 4f 5/2 orbitals of metallic gold (Au0).

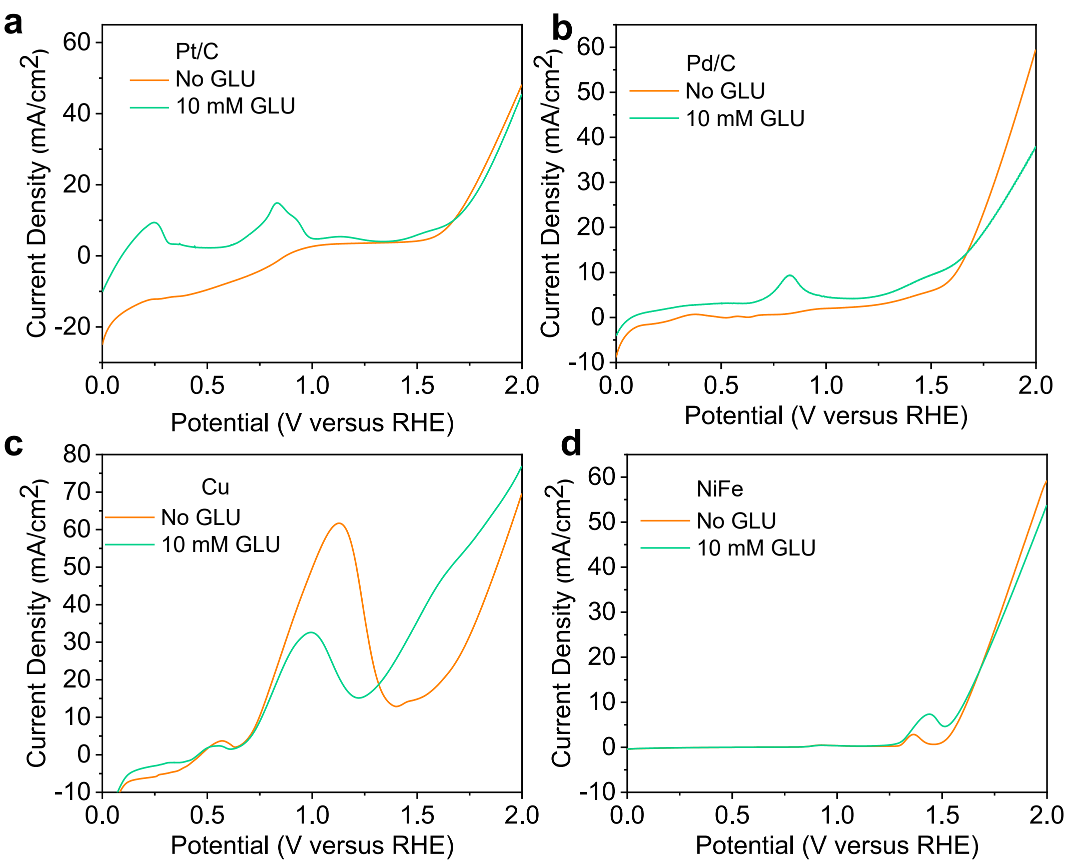


**Figure S14.** LSV curves of catalysts in 1 M KOH, with (green) and without (yellow) 10 mM GLU. (a) Pt/C, (b) Pd/C, (c) Cu, and (d) NiFe.

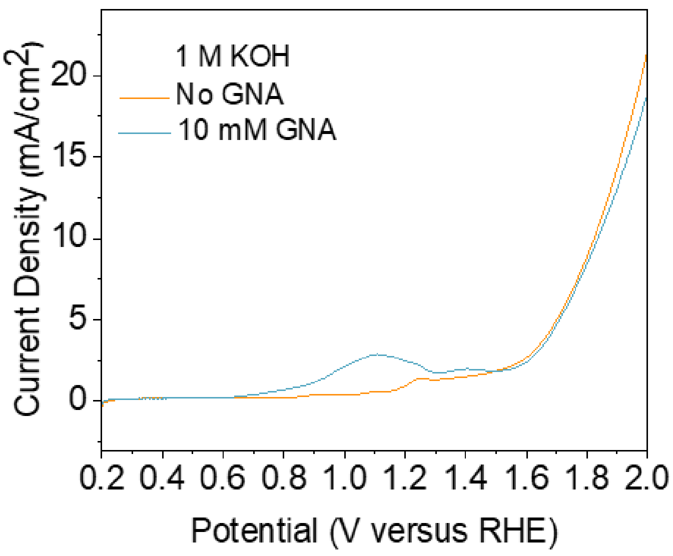


**Figure S15.** LSV curves of Au/C electrode in 1 M KOH, with (blue) and without (yellow) 10 mM GNA.

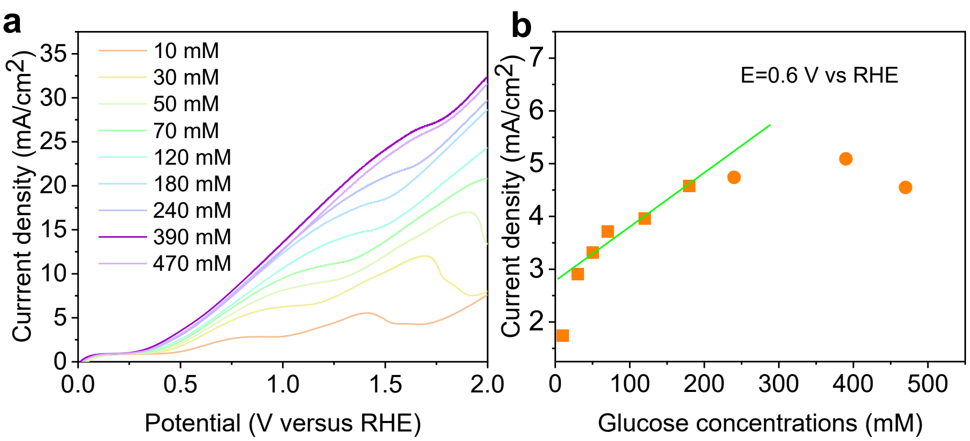


**Figure S16.** Electrochemical behaviors of the oxidation of GLU with different concentrations and Au/C as electrocatalyst. (a) LSV profiles of the oxidation of GLU with different concentrations; (b) the calibration curves of the GLU concentrations with the current densities in potential of 0.6 V.

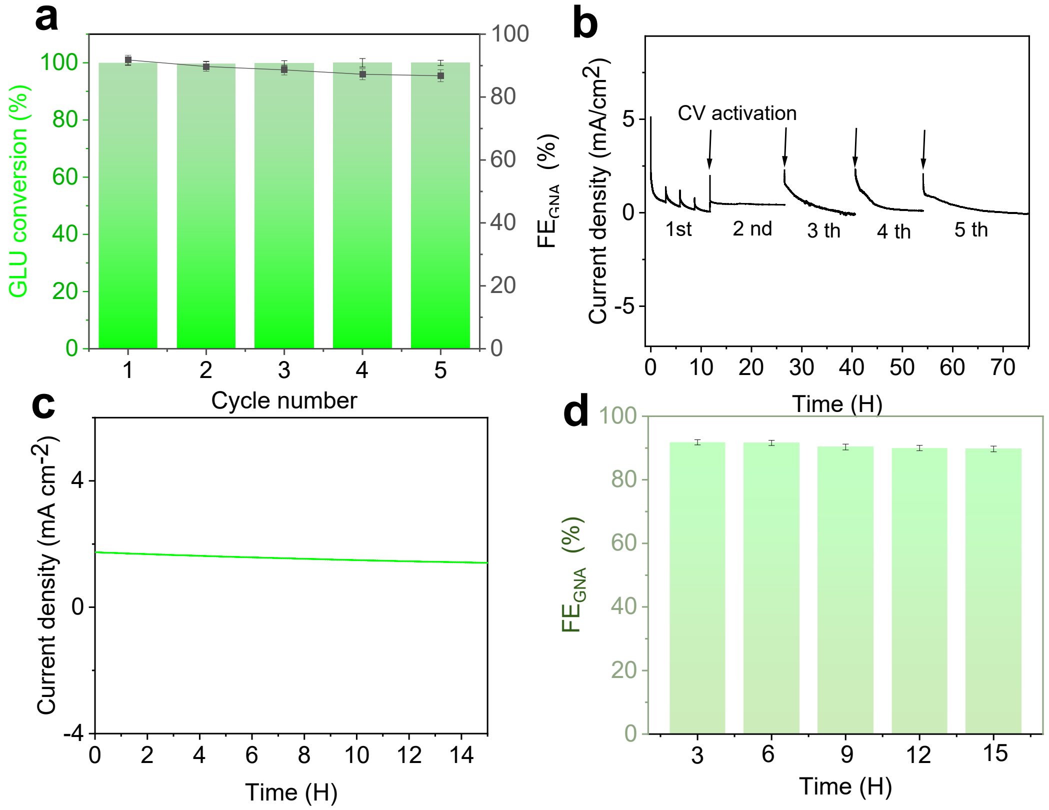


**Figure S17.** Stability test of Au/C. (a) GLU conversion and FE $_{GNA}$ of the Au/C electrode in the cycle used for GLU oxidation at 0.6 V and (b) Chronoamperometric study (current density as a function of time), the arrow indicates CV activation. (c) Long-term stability of Au/C catalyst for GLU oxidation at 0.6 V with fresh 10 mM GLU solution in a flow cell and (d) $FE_{GNA}$ in the chronoamperometry process. Passivation can occur during the operation of working electrodes. To reactivate the electrode when the current decreases to the 0 mA threshold, 10 cyclic voltammetry cycles are performed from 0.2 to 0.8 V at 50 mV/s, effectively restoring its activity.

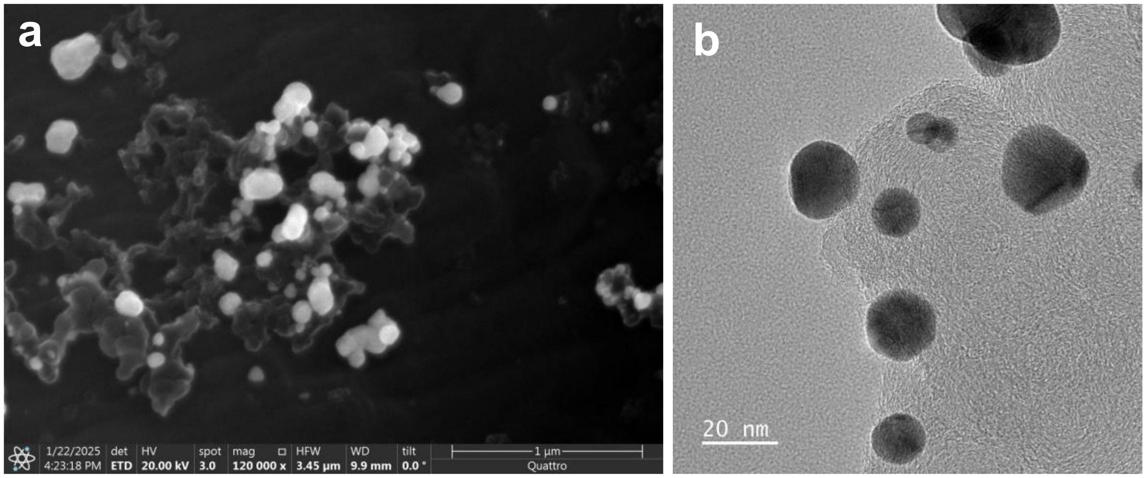


**Figure S18.** (a) SEM and (b) TEM spectra of the Au/C electrode after reused.

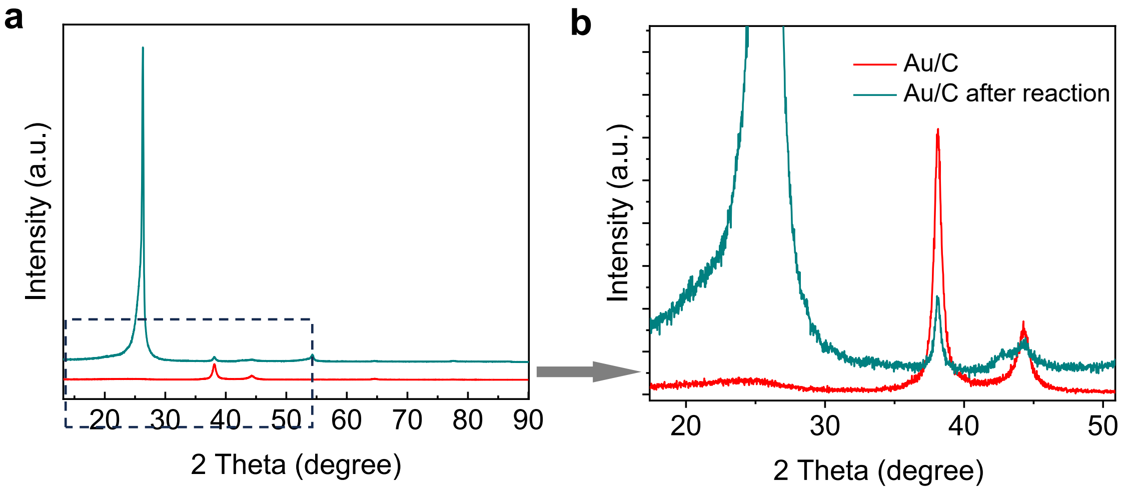


**Figure S19.** (a) XRD spectra and (b) enlarged region of the Au/C electrode after reused.

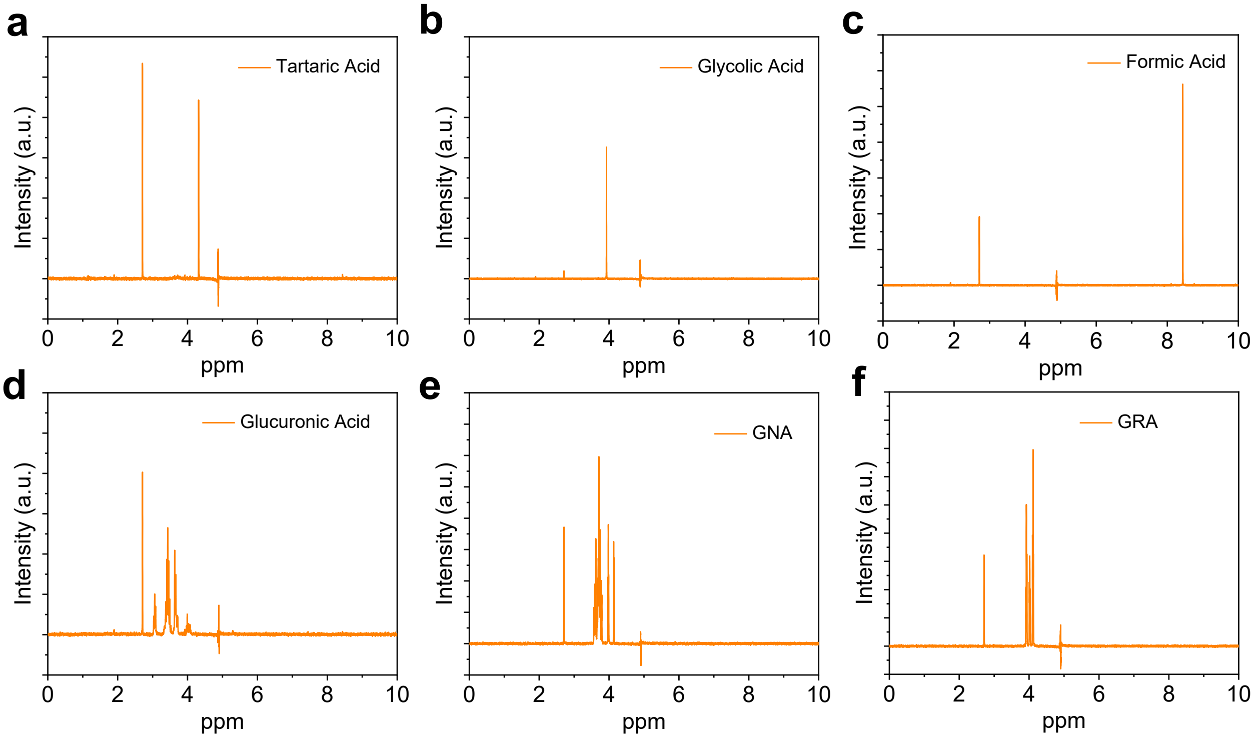


**Figure S20.** $^1$H spectra of pure (a) tartaric acid, (b) glyconlic acid, (c) HCOOH, (d) glucuronic acid, (e) GNA, and (f) GRA

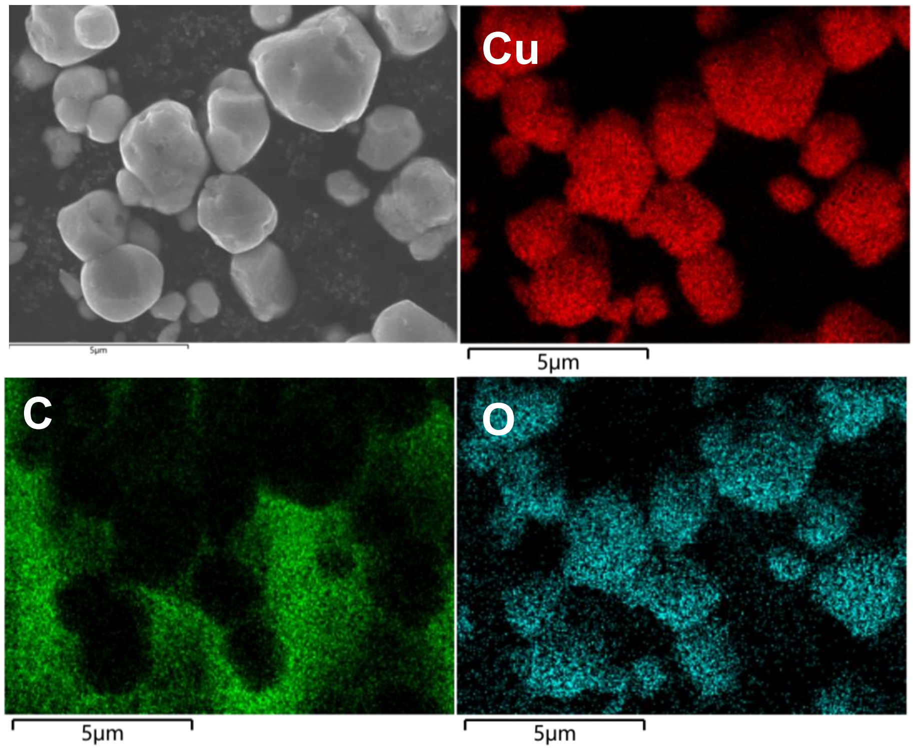


**Figure S21.** SEM and EDS pattern of the $Cu_2O$.

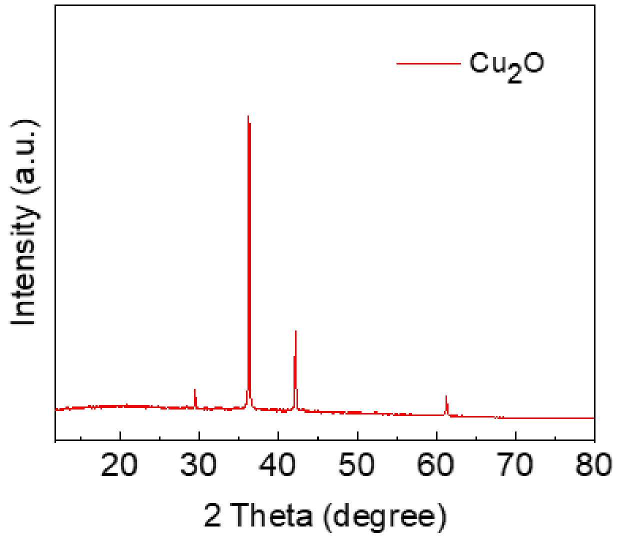


**Figure S22.** XRD pattern of $Cu_2O$

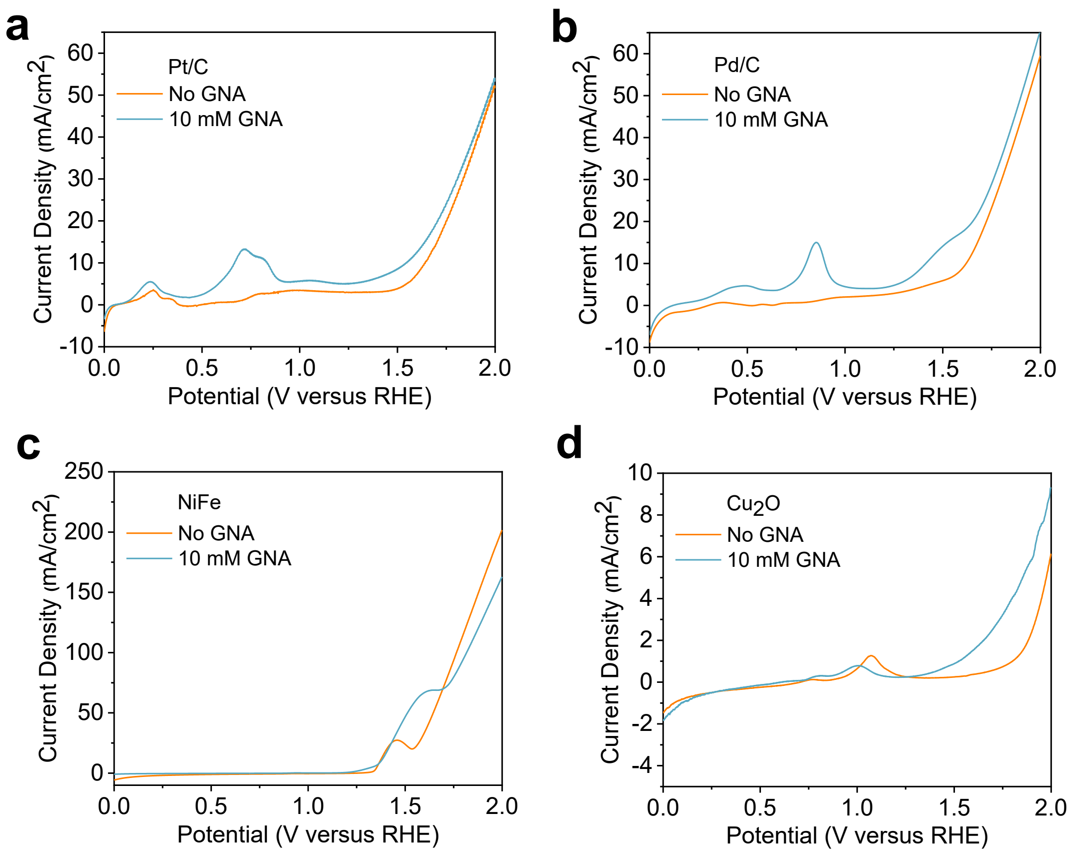


**Figure S23.** LSV curves of catalysts in 1 M KOH, with (blue) and without (red) 10 mM GNA. (a) Pt/C, (b) Pd/C, (c) NiFe and (d) $Cu_2O$.

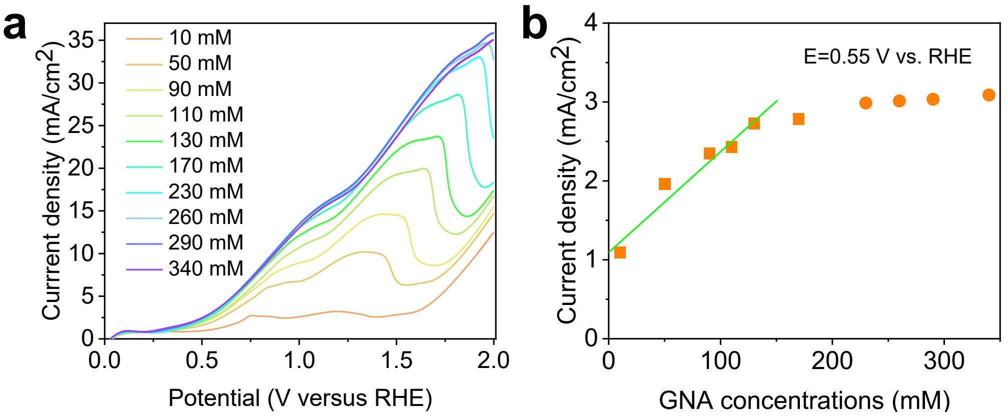


**Figure S24.** Electrochemical behaviors of the oxidation of GNA with different concentrations and AuPt/C as electrocatalyst. (a) LSV profiles of the oxidation of GNA with different concentrations; (b) The calibration curves of the GNA concentrations with the current densities in potential of 0.55 V vs RHE.

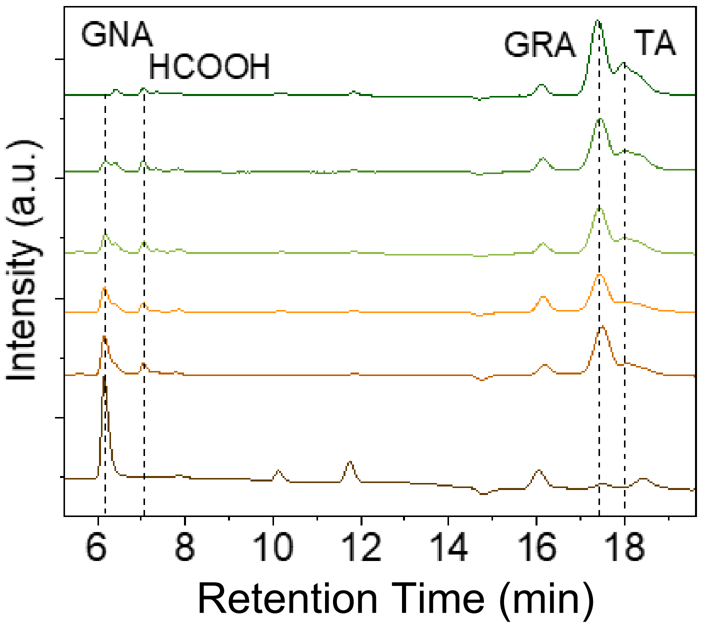


**Figure S25.** HPLC chromatograms for the GNA oxidation at different reaction times on AuPt/C catalyst (initial GNA concentration: 10 mM)

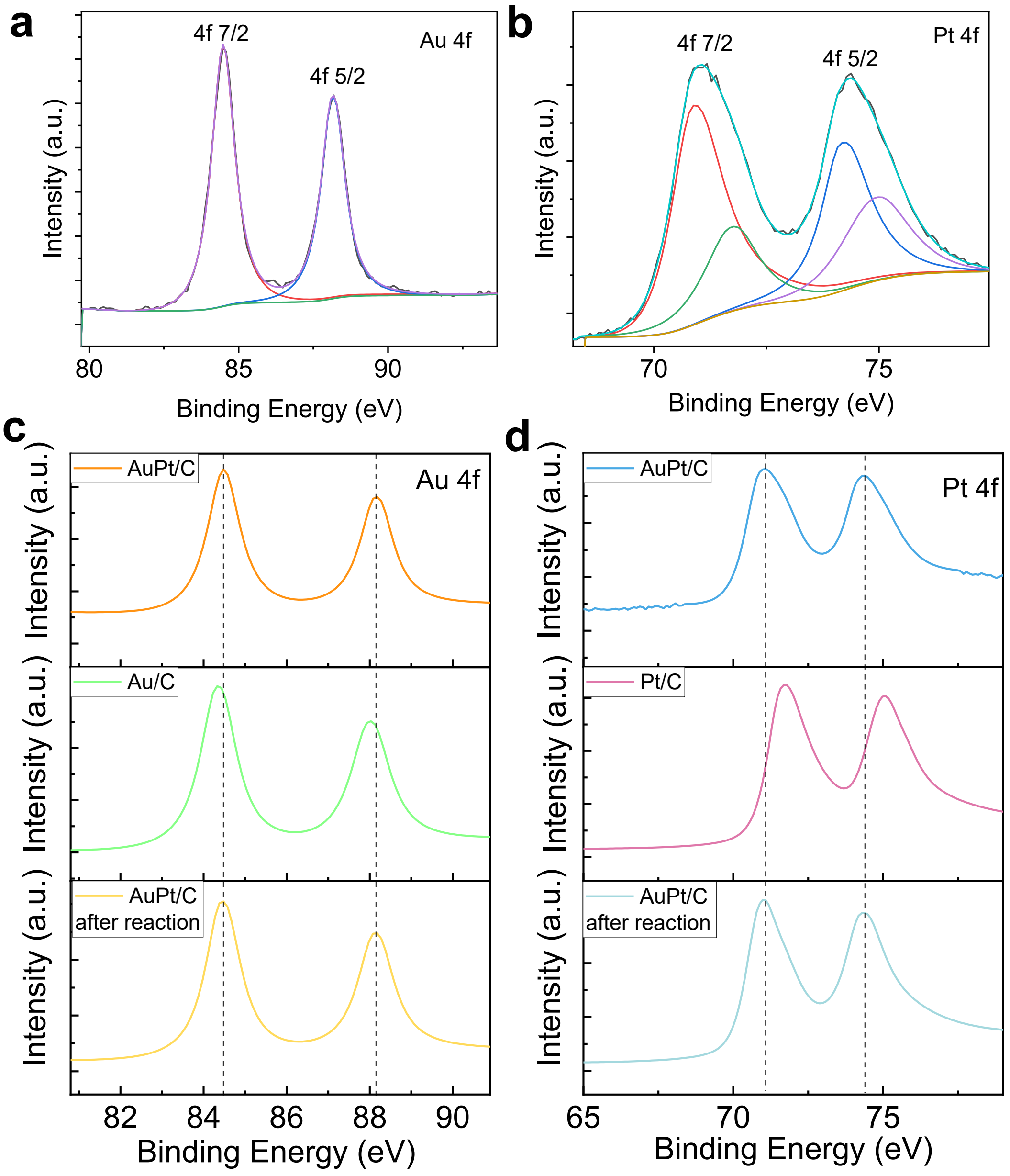


**Figure S26.** XPS of samples. (a) Au 4f and (b) Pt 4f spectra of the AuPt/C. Comparison of (c) Au 4f and (d) Pt 4f XPS pattern before and after the electrooxidation reaction.

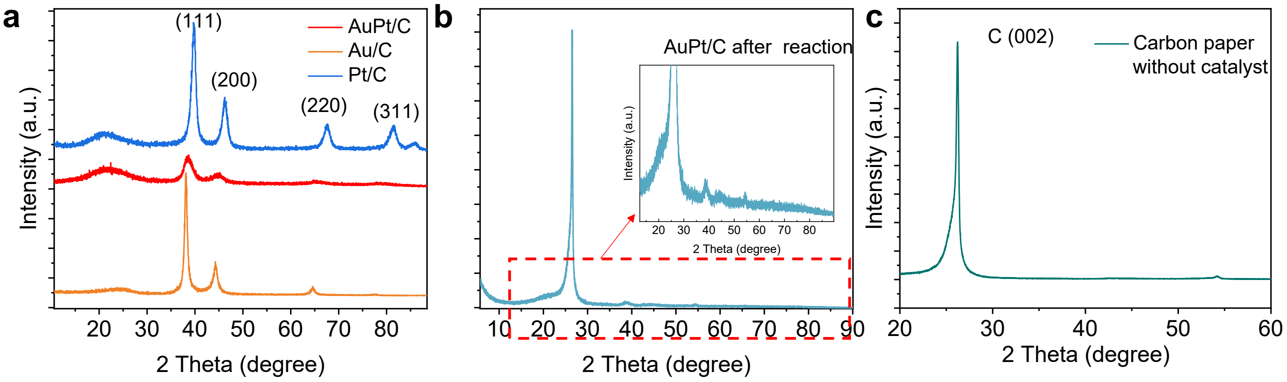


**Figure S27.** (a) XRD pattern of the AuPt/C, Au/C, and Pt/C, (b) AuPt/C after reaction and (c) carbon paper. After the reaction, the characteristic peaks of (111) and (200) of the AuPt/C catalyst remained unchanged. The peak at ~26° comes from the carbon paper substrate, as confirmed in Figure S27c showing the XRD pattern of the carbon paper.

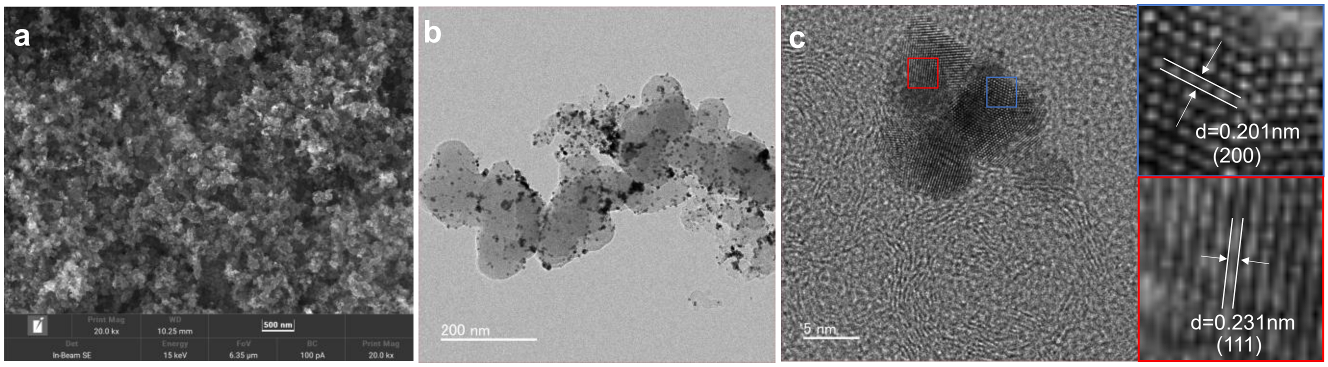


**Figure S28.** (a) SEM and (b-c) TEM pattern of the AuPt/C.

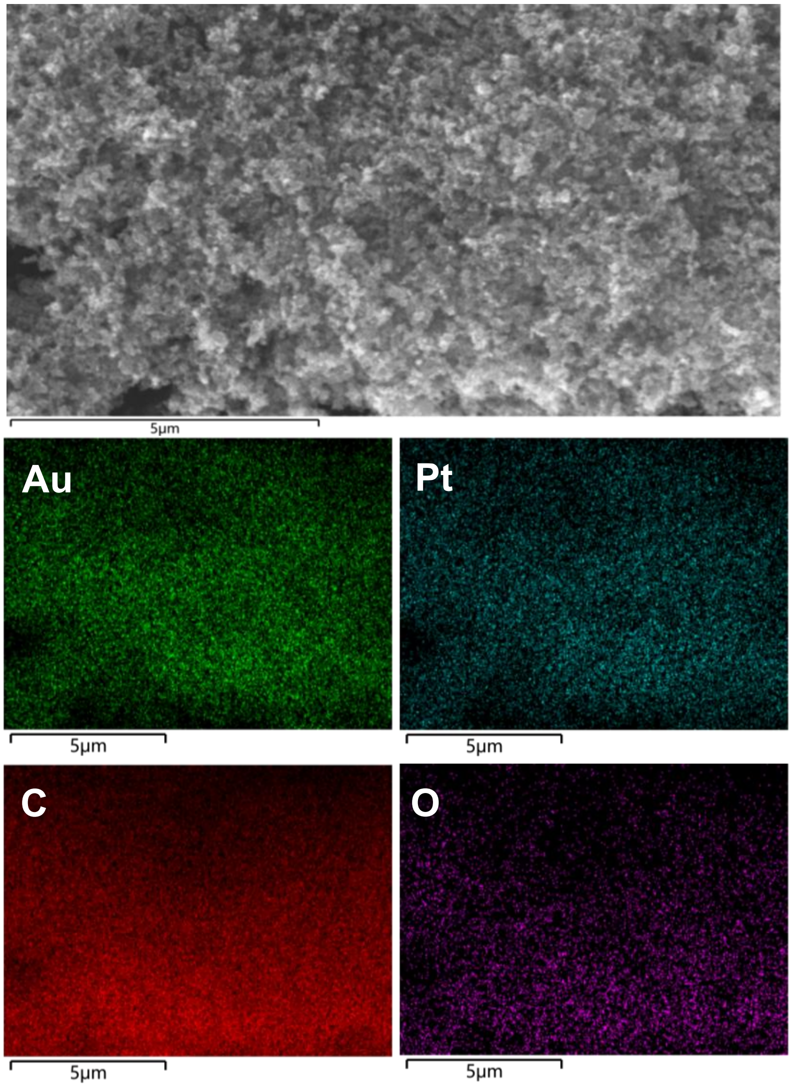


**Figure S29.** SEM-EDS elemental mapping of the AuPt/C electrode catalyst. The EDX pattern confirms the uniform distribution of Pt, Au and C.

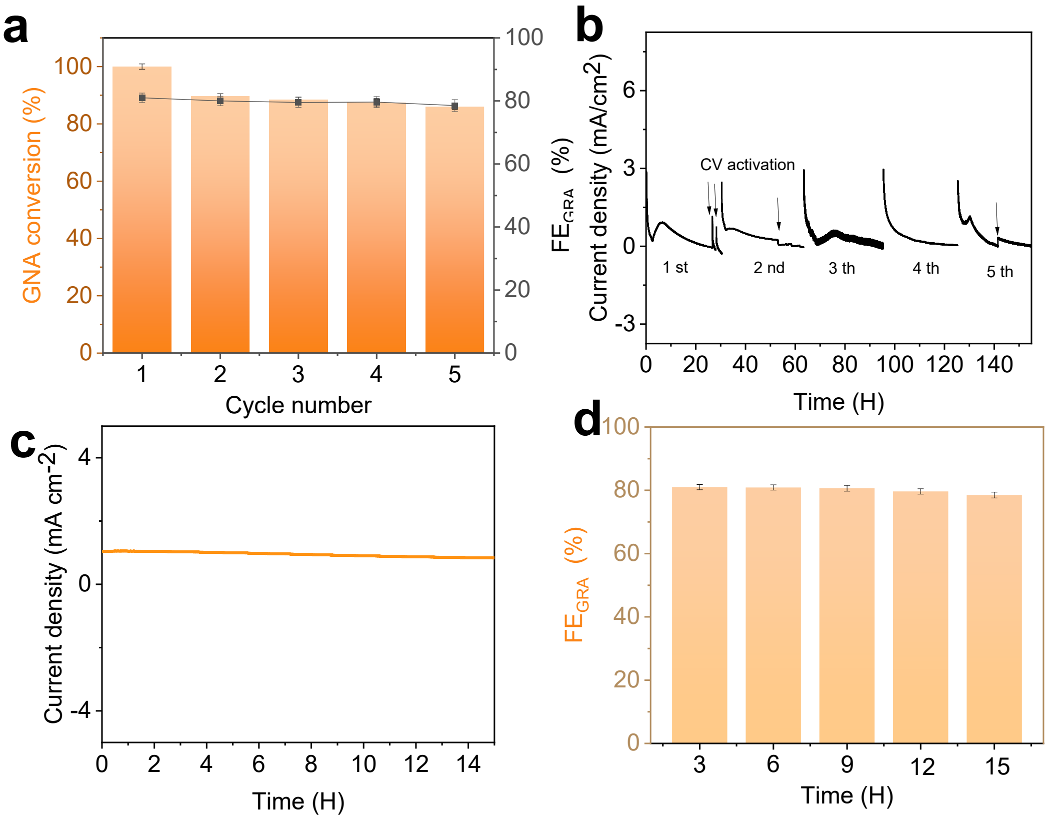


**Figure S30.** Stability testing of AuPt/C. (a) GNA conversion and GRA yield of the AuPt/C electrode in the cycle used for GNA oxidation at 0.55 V and (b) Chronoamperometric study (current density as a function of time), the arrow indicates CV activation. (c) Long-term stability of AuPt/C catalyst for GNA oxidation at 0.55 V (A fresh 10 mM GNA solution was continuously passed through the reaction process) and (d) $FE_{GRA}$ in the chronoamperometry process.

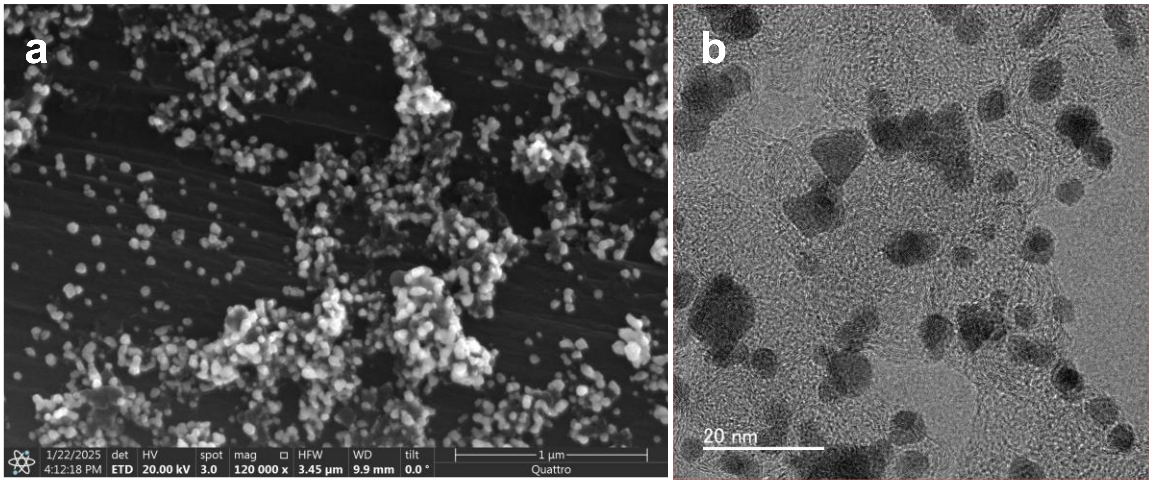


**Figure S31.** (a) SEM and (b) TEM spectra of the AuPt/C electrode after reused.

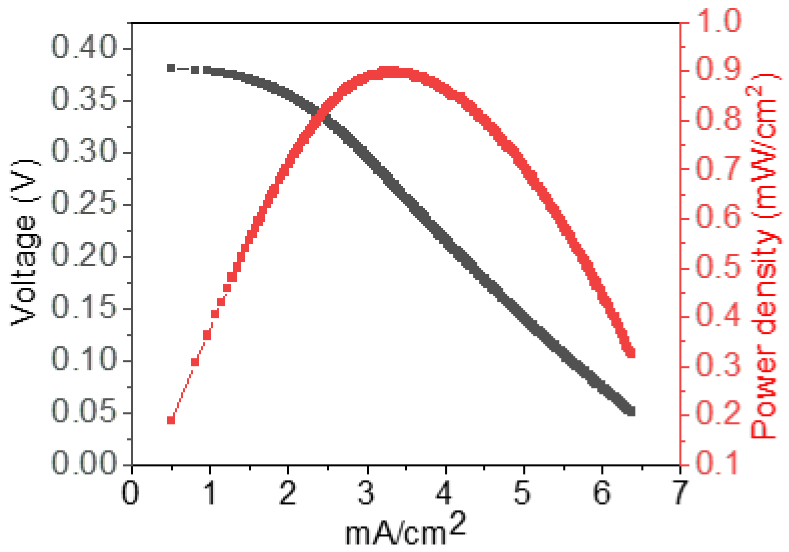


**Figure S32.** Polarization curve of the GLU fuel cell.

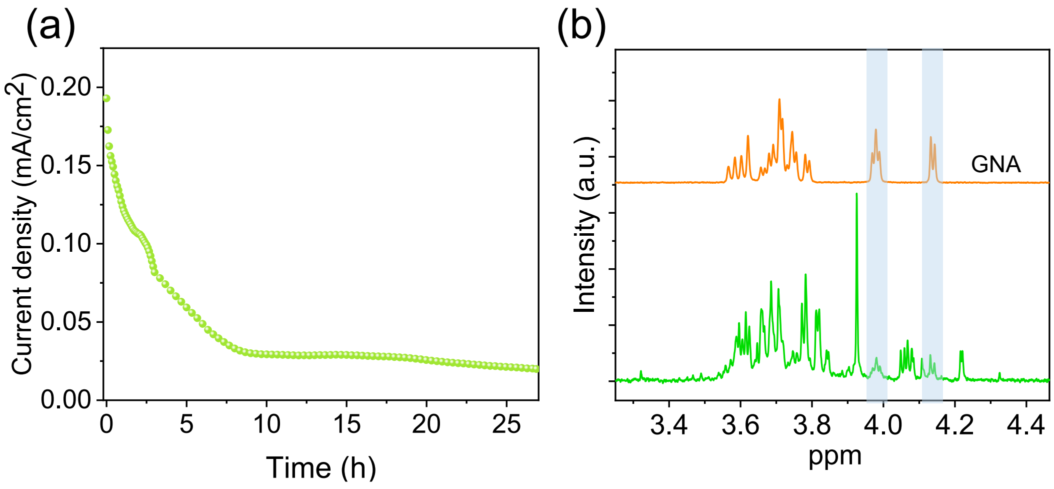


**Figure S33.** (a) Curve of current versus time during constant voltage (0.37V) discharge of GLU fuel cell, (b) $^{1}H$ spectra of the electrolyte after complete discharge (green) and pure GNA (origin).

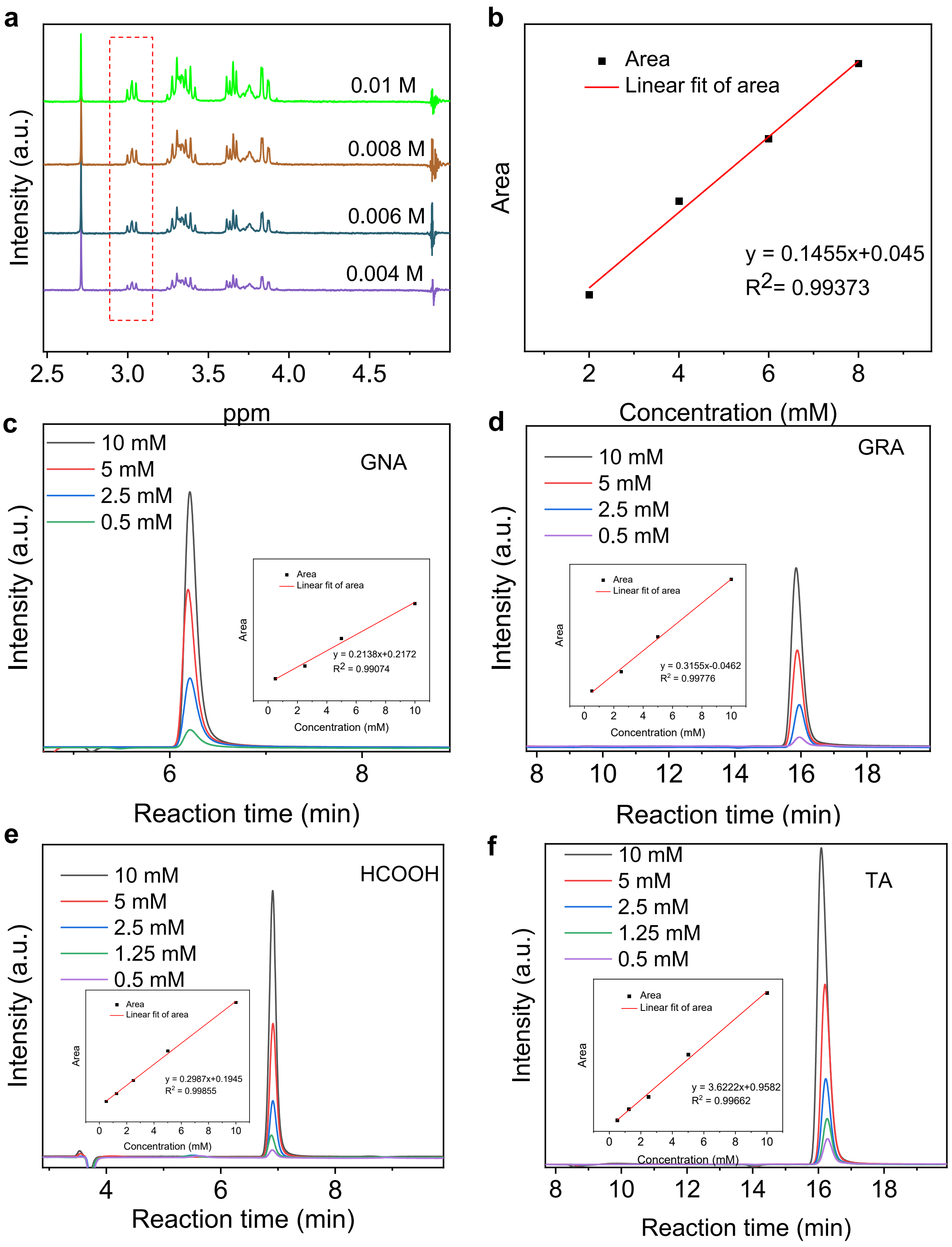


**Figure S34.** (a) $^{1}$H spectra of pure GLU, (b) Calibration curves of the GLU. HPLC measurements of pure (c) GNA, (d) GRA, (e) HCOOH and (f) TA. The inset is the Calibration curves of the HPLC.

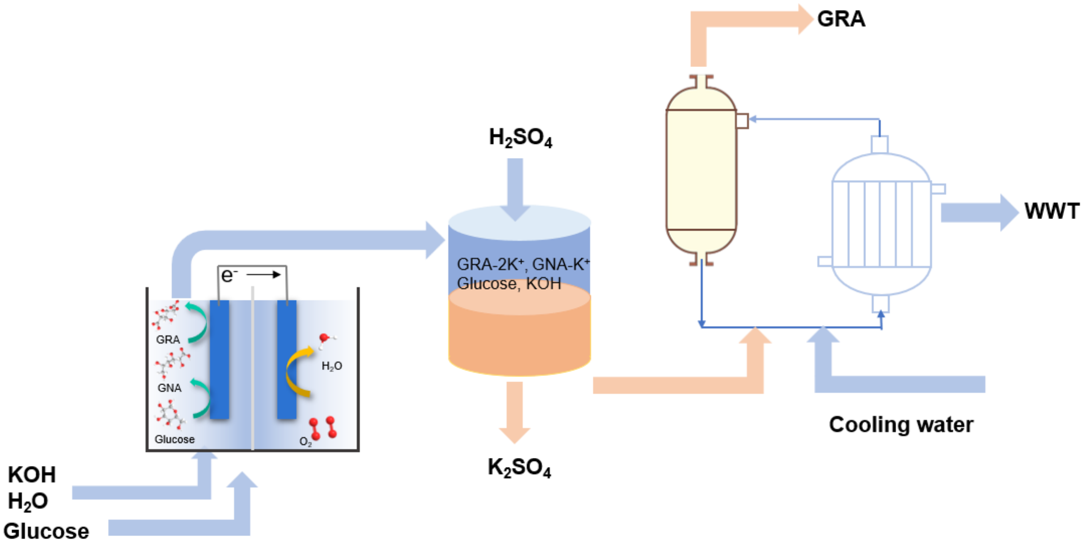


**Figure S35.** Simplified process flow diagram of the two-step tandem electrocatalytic GLU oxidation process.

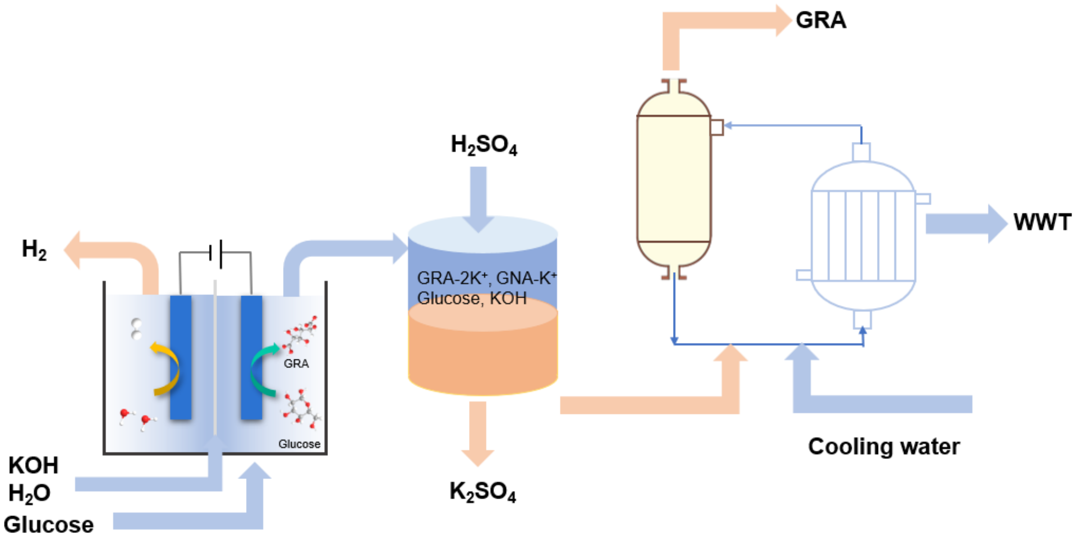


**Figure S36.** Simplified process flow diagram of the one step electrocatalytic GLU oxidation process.

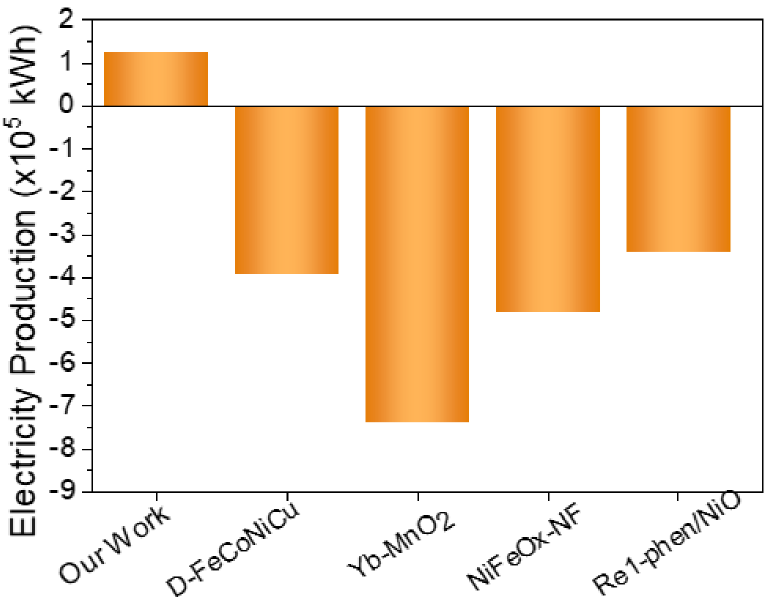


**Figure S37.** Comparison of electricity consumption for the production of GRA (1000 ton of GRA per year) by two-step tandem system GLU oxidation and other literature (coupled with HER).

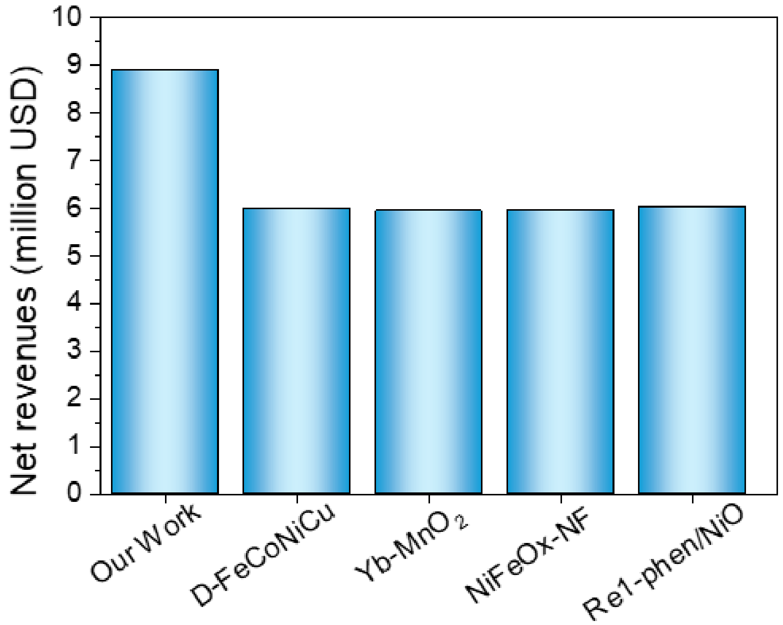


**Figure S38.** Comparison of net revenues from different catalysts producing 1000t of GRA. After deducting costs such as sum raw materials costs, sum utilities, and total operating costs, the net revenues of our series two-step system are significantly greater than those of other one-step methods, highlighting our economic benefits.

**Table S1.** Catalysts and process conditions for obtaining GNA/GRA in a one-step reaction from GLU.

| Catalyst | Products | Electrolyte | Voltage (V) | FE | Stability | Ref. |
|---|---|---|---|---|---|---|
| Cu（I）/Cu（II） | GNA | 1 M KOH+0.1 M GLU | 1.20 | 98.7% | 10 h | [2] |
| D-FeCoNiCu-LDH/NF | GRA | 1 M KOH+0.1 M GLU | 1.22 | 90% | 50h | [3] |
| $Re_1$-phen/NiO | GRA | 1.0 M KOH with 0.1 M GLU | 1.30 | 94% | 18h | [4] |
| Yb-$MnO_2$ | GRA | 0.05 M $H_2SO_4$+0.1 M GLU | 1.558 | 84.6% | 50h | [5] |
| $NiFeO_x$-NF//$NiFeN_x$-NF | GRA | 1 M KOH+0.5 M GLU | 1.30 | 87% | Five cycles | [6] |
| Au/C<br>AuPt/C | GNA<br>GRA | 1 M KOH+0.01 M GLU | 0.60V for GNA<br>0.55V for GRA | 91.8% for GNA,<br>81% for GRA | 15h<br>15h | Our work |

**Table S2.** List of the economic parameters and assumptions for a 1.0 kton/year process to produce GRA.

| Economic parameters | | Values |
|---|---|---|
| GLU price ($ per ton) [a] | | 444 |
| GNA price ($ per ton) [a] | | 1500 |
| GRA price ($ per ton) [a] | | 12000 |
| KOH price ($ per ton) [a] | | 820 |
| Process water price ($ per ton) [b] | | 0.22 |
| $H_2$ ($ per ton)[a] | | 1800 |
| $H_2SO_4$[a] | | 143 |
| $K_2SO_4$[a] | | 400 |
| Electricity price ($ per kWh)[a] | | 0.055 |
| 20 wt% AuC ($ per Kg)[c] | Recyclable (50%) | 12000 |
| 20 wt% AuPtC ($ per Kg)[c] | Recyclable (50%) | 9500 |
| Carbon paper ($ per $m^2$)[c] | Recyclable (50%) | 66.37 |

[a] Khan, M.A. et al. Techno-economic analysis of a solar-powered biomass electrolysis pathway for coproduction of hydrogen and value-added chemicals. Sustainable Energy & Fuels 4, 5568-5577 (2020).

b Government set industrial water price (http://tazlh.zjzwfw.gov.cn/art/2014/6/16/art-30833-28099.html)

[c] Taken from online market data (https://item.taobao.com/item)

**Table S3.** Equipment list and installation costs for the GRA production via two steps electrocatalytic oxidation process.

| Equipment ID | Equipment name | Amount | Scale cost ($) | Installation factor | Installed cost ($) | Notes |
|---|---|---|---|---|---|---|
| EM | Material mixer | 2 | 26226 | 1.68 | 44060 | Corrosion resistance |
| EP | Centrifugal pump | 2 | 125200 | 2.43 | 304236 | Corrosion resistance |
| $EE_1$ | Electrolyzer | 10 | 1052300 | 1.12 | 1178576 | Corrosion resistance |
| BC | Boost Converter | 10 | 972 | 1.12 | 10880 | |
| ED | Electronic decoupler | 10 | 1428 | 1.12 | 16000 | |
| $EE_2$ | Electrolyzer | 10 | 1052300 | 1.12 | 1178576 | Corrosion resistance |
| ER | Reactor | 1 | 120240 | 1.42 | 171943 | Corrosion resistance |
| EC | Crystallizer | 2 | 557460 | 2.58 | 1438246 | - |
| EH | Heat exchanger | 2 | 141540 | 1.26 | 178340 | Cooling |
| ECE | Centrifugal machine | 2 | 482660 | 1.82 | 878441 | - |
| ED | Drying machine | 2 | 563400 | 1.48 | 833832 | - |

**Table S4.** Equipment list and installation costs for the GRA/GNA production via one step electrocatalytic oxidation process.

| Equipment ID | Equipment name | Amount | Scale cost ($) | Installation factor | Installed cost ($) | Notes |
|---|---|---|---|---|---|---|
| EM | Material mixer | 2 | 26226 | 1.68 | 44060 | Corrosion resistance |
| EP | Centrifugal pump | 2 | 125200 | 2.43 | 304236 | Corrosion resistance |
| EE | Electrolyzer | 20 | 2104600 | 1.12 | 2357152 | Corrosion resistance |
| ER | Reactor | 1 | 120240 | 1.42 | 171943 | Corrosion resistance |
| EC | Crystallizer | 2 | 557460 | 2.58 | 1438246 | - |
| EH | Heat exchanger | 2 | 141540 | 1.26 | 178340 | Cooling |
| ECE | Centrifugal machine | 2 | 482660 | 1.82 | 878441 | - |
| ED | Drying machine | 2 | 563400 | 1.48 | 833832 | - |

**Table S5.** Total direct costs, indirect cost and capital investment for a 1 kton/year process to produce GRA

| | Notes | Two-step tandem | One step |
|---|---|---|---|
| GRA/GNA production | - | 2884446 | 2705448 |
| GRA/GNA separation and purification | - | 3500802 | 3500802 |
| Products storage | - | 560078 | 546678 |
| Heat exchange | Heating and cooling | 1049040 | 1023943 |
| WWTs | Wastewater treatments | 1601718 | 1563398 |
| Others | utility | 779006 | 760369 |
| **Total installed cost** | | **10375090** | **10100639** |
| Warehouse (4.0% of ISBL) | Inside-battery-limits (ISBL): cost for products production, separation and purification | 255409 | 248250 |
| Site Development (9.0% of ISBL) | | 574672 | 558563 |
| Additional Piping (4.5% of ISBL) | | 287336 | 279281 |
| **Total Direct Cost (TDC)** | Total installed cost +Warehouse + Site Development + Additional Piping | **11492508** | **11186733** |
| Prorateable Expenses | 10% of TDC | 1149250 | 1118673 |
| Field Expenses | 10% of TDC | 1149250 | 1118673 |
| Home Office & Construction | 20% of TDC | 2298501 | 2237347 |
| Project Contingency | 10% of TDC | 1149250 | 1118673 |
| Other Costs (Start-Up, Permits, etc.) | 10% of TDC | 1149250 | 1118673 |
| **Total Indirect Cost (TIC)** | **-** | **6895505** | **6712040** |
| **Fixed Capital Investment (FCI)** | Including the capital cost growth (0.64) | **11768328** | **11455214** |
| Land | - | 143534 | 139715 |
| Working Capital | 5% of FCI | 588416 | 572761 |
| **Total Capital Investment (TCI)** | **FCI + Land + Working capital** | **12500279** | **12167690** |

**Table S6.** Operating costs, and revenue streams of the electrocatalytic and non-electrocatalytic GLU oxidation processes for a 1 kton/year process to produce GRA.

| | Notes | Two-step tandem system ($ year$^{-1}$) | One step ($ year$^{-1}$) |
|---|---|---|---|
| GLU | 10 | 498240 | 532800 |
| KOH | - | 312420 | 333084 |
| $H_2SO_4$ | - | 475332 | 507650 |
| Catalysts cost | Recyclable (50%) | 4123 | 100 |
| Process water | - | 149160 | 152626 |
| Carbon paper | Recyclable | 101 | - |
| **Sum Raw materials costs** | | **1439376** | **1526260** |
| Steam | - | 427137 | 226337 |
| Cooling water | | 20629 | 126274 |
| Electricity | - | - | 1230178 |
| **Sum Utilities** | Steam + Cooling water + Electricity | **447766** | **1582788** |
| Fixed operating costs | - | 190343 | 252476 |
| Other operating costs | - | 13085 | 17356 |
| **Total Operating costs** | Raw materials costs + Utilities + Fixed operating costs + Other operating costs | **2090570** | **3378881** |
| $K_2SO_4$ sale | - | 238000 | 90204 |
| GRA sale | | 12000000 | 12000000 |
| GNA sale | - | - | - |
| $H_2$ sale | - | - | 55800 |
| Electricity sale | | 6829 | - |
| **Revenues (R)** | | **12244829** | **12146004** |

**References**

1. Lu, X. & Zhao, C. Electrodeposition of hierarchically structured three-dimensional nickel–iron electrodes for efficient oxygen evolution at high current densities. Nat. Commun. 6, 6616 (2015).

2. Zhang, Y. et al. Coupling Glucose-Assisted Cu(I)/Cu(II) Redox with Electrochemical Hydrogen Production. Adv. Mater. 33, 2104791 (2021).

3. Wu, X. et al. Multi-site catalysis of high-entropy hydroxides for sustainable electrooxidation of glucose to glucaric acid. Energ. Environ. Sci. 17, 3042-3051 (2024).

4. Jiang, X. et al. "Suspended" Single Rhenium Atoms on Nickel Oxide for Efficient Electrochemical Oxidation of Glucose. J. Am. Chem. Soc. 147, 4886-4895 (2025).

5. Li, J. et al. Efficiently coupled glucose oxidation for high-value D-glucaric acid with ultradurable hydrogen via Mn(III) in acidic solution. Nano Res. 16, 10748-10755 (2023).

6. Liu, W.-J. et al. Efficient electrochemical production of glucaric acid and $H_2$ via glucose electrolysis. Nat. Commun. 11, 265 (2020).
